# Integrated network pharmacology analysis, molecular docking, LC-MS analysis and bioassays revealed the potential active ingredients and underlying mechanism of *Scutellariae radix* for COVID-19

**DOI:** 10.3389/fpls.2022.988655

**Published:** 2022-09-15

**Authors:** Jiazheng Liu, Jieru Meng, Runfeng Li, Haiming Jiang, Lu Fu, Ting Xu, Guo-Yuan Zhu, Wei Zhang, Jin Gao, Zhi-Hong Jiang, Zi-Feng Yang, Li-Ping Bai

**Affiliations:** ^1^State Key Laboratory of Quality Research in Chinese Medicine, Macau Institute for Applied Research in Medicine and Health, Guangdong-Hong Kong-Macao Joint Laboratory of Respiratory Infectious Disease, Macau University of Science and Technology, Macao, Macao SAR, China; ^2^State Key Laboratory of Respiratory Disease, National Clinical Research Center for Respiratory Disease, Guangzhou Institute of Respiratory Health, The First Affiliated Hospital of Guangzhou Medical University, Guangzhou, Guangdong, China; ^3^Increasepharm (Hengqin) Institute Co., Ltd., Zhuhai, Guangdong, China; ^4^Guangzhou Key Laboratory for Clinical Rapid Diagnosis and Early Warning of Infectious Diseases, Guangzhou, Guangdong, China

**Keywords:** *Scutellariae radix*, LC-MS analysis, network pharmacology, COVID-19, anti-inflammatory, anti-infective

## Abstract

*Scutellariae radix* (“Huang-Qin” in Chinese) is a well-known traditional herbal medicine and popular dietary supplement in the world, extensively used in prescriptions of TCMs as adjuvant treatments for coronavirus pneumonia 2019 (COVID-19) patients in China. According to the differences in its appearance, *Scutellariae radix* can be classified into two kinds: ZiQin (1∼3 year-old *Scutellariae baicalensis* with hard roots) and KuQin (more than 3 year-old *S. baicalensis* with withered pithy roots). In accordance with the clinical theory of TCM, KuQin is superior to ZiQin in cooling down the heat in the lung. However, the potential active ingredients and underlying mechanisms of *Scutellariae radix* for the treatment of COVID-19 remain largely unexplored. It is still not clear whether there is a difference in the curative effect of ZiQin and KuQin for the treatment of COVID-19. In this research, network pharmacology, LC-MS based plant metabolomics, and *in vitro* bioassays were integrated to explore both the potential active components and mechanism of *Scutellariae radix* for the treatment of COVID-19. As the results, network pharmacology combined with molecular docking analysis indicated that *Scutellariae radix* primarily regulates the MAPK and NF-κB signaling pathways *via* active components such as baicalein and scutellarin, and blocks SARS-CoV-2 spike binding to human ACE2 receptors. *In vitro* bioassays showed that baicalein and scutellarein exhibited more potent anti-inflammatory and anti-infectious effects than baicalin, the component with the highest content in *Scutellariae radix*. Moreover, baicalein inhibited SARS-CoV-2’s entry into Vero E6 cells with an IC_50_ value of 142.50 μM in a plaque formation assay. Taken together, baicalein was considered to be the most crucial active component of *Scutellariae radix* for the treatment of COVID-19 by integrative analysis. In addition, our bioassay study revealed that KuQin outperforms ZiQin in the treatment of COVID-19. Meanwhile, plant metabolomics revealed that baicalein was the compound with the most significant increase in KuQin compared to ZiQin, implying the primary reason for the superiority of KuQin over ZiQin in the treatment of COVID-19.

## Introduction

**FIGURE 1:**
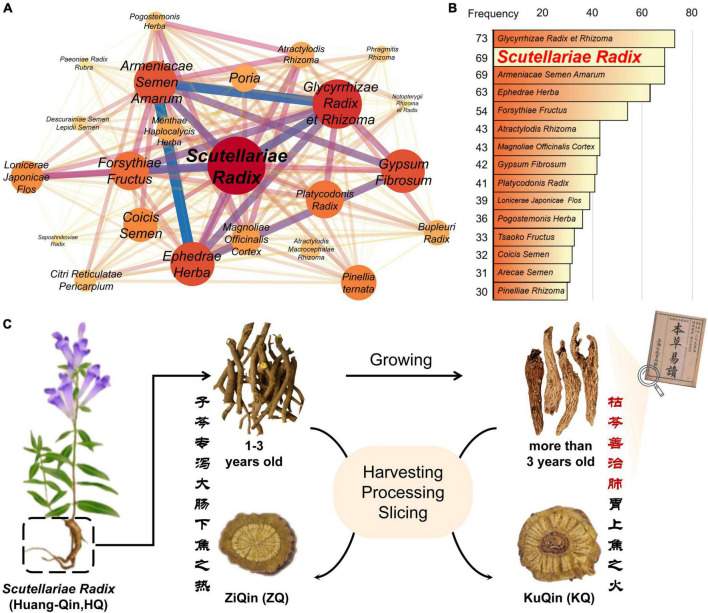
**(A)** Analysis of the association rules of traditional Chinese herbal medicines used in adjuvant treatment of COVID-19. **(B)** Frequency analysis of single Chinese herbal medicine. **(C)** Schematic diagram of the source of ZiQin and KuQin. The classical Chinese shown in the picture means “400 years ago, ancient Chinese medicine books recorded that KuQin was good at cooling down the heat in the lung and stomach, and ZiQin was good at reducing the large-intestinal heat”.

Coronavirus disease 2019 (COVID-19) pandemic, caused by the severe acute respiratory syndrome coronavirus 2 (SARS-CoV-2), has recently resulted in a global public health crisis, and posed a huge threat and challenge to the public healthcare system. In China, traditional Chinese medicines (TCMs) have made great contributions in the battle against COVID-19 (Li L. et al., 2021; Lyu et al., 2021; Zhao et al., 2021), in combination with Western medicines. Based on a statistical analysis of usage frequency in the compositions of the TCMs prescriptions for COVID-19 in 34 provincial areas of China, it was found that the dried root of *Scutellaria baicalensis Georgi* (named as “Huang-Qin” in Chinese) is one of the most commonly used TCMs for adjuvant treatment of COVID-19 (Figures 1A,B). Due to its anti-inflammatory and anti-infectious activities, *Scutellariae radix* has been used by Chinese people for the treatment of diseases including upper respiratory tract infection and enteritis over more than 3,000 years (Dinda et al., 2017; Liao et al., 2021). Moreover, *Scutellariae radix* is also a popular functional food and dietary supplement commonly used internationally (Delerue et al., 2021).

Based on the different appearance and the theory of traditional Chinese medicine, *Scutellariae radix* is divided into two types of commercial products specification. One is “ZiQin” with complete and hard xylem from 1 ∼ 3 year-old *S. baicalensis*, and the other is “KuQin” with withered and rotten xylem from more than 3 year-old *S. baicalensis* (Figure 1C and Supplementary Figure 1) (Cao et al., 2019; Ren et al., 2021). According to the clinical experience of traditional Chinese medicine, KuQin is superior to ZiQin in the treatment of pneumonia, and ZiQin has a better effect than KuQin in the treatment of enteritis (Ren et al., 2021). Recently, there have been a few reports on the anti-COVID-19 effects of the extract of *Scutellariae radix* (Song et al., 2020, 2021; Gao et al., 2021; Liu et al., 2021). However, it remains unexplored for the potential active ingredients and underlying mechanism of *Scutellariae radix* for the treatment of COVID-19. It is still unclear whether there is a difference in the curative effect of KuQin and ZiQin for the treatment of COVID-19. This study explored potential active components and anti-COVID-19 mechanism, anti-inflammatory and anti-infectious effects of both ZiQin and KuQin based on an integrated approach of network pharmacology analysis, molecular docking, LC-MS based plant metabolomics, and pharmacological validation (Deng et al., 2020; Pan et al., 2020; Yang et al., 2020; Li Z. T. et al., 2021; Zhou et al., 2021; Zhang et al., 2022). The chemical difference and potential anti-COVID-19 effect of ZiQin and KuQin were also compared. The findings of this study were aimed to provide a scientific support and valuable guidance to the proper usage of *Scutellariae radix* for COVID-19.

## Materials and methods

### Reagents and standards

Deionized water was prepared by using a Millipore water purification system (Merck, Hong Kong, China). LC-MS grade acetonitrile, methanol, and formic acid were bought from Anaqua Global International Inc., Limited (United States). LPS (*Escherichia coli* 0127: B8) and dexamethasone were obtained from Sigma Chemical Co. (St. Louis, MO, United States). Chemical standards for UHPLC-Q-TOF-MS analyses were purchased from Chengdu Must Bio-Technology Co., Ltd. (Apigenin ≥ 98%, Baicalein ≥ 98%, Chrysin ≥ 98%, scutellarein ≥ 98%, Wogonin ≥ 98%, Oroxylin A ≥ 98%, and Baicalin ≥ 98% in purity), and Innochem (Isoscutellarein ≥ 95%, and Wogonoside ≥ 98% in purity), respectively.

### Sample collection of *Scutellariae radix*

The dried roots of *S. baicalensis Georgi* were collected from provinces of Inner Mongolia, Shanxi and Hebei of China during September in 2020 to March in 2021, and authenticated by Dr. Bai Li-Ping (State Key Laboratory of Quality Research in Chinese Medicine, Macau University of Science and Technology, Taipa, Macau, China). The detailed information of samples was shown in Supplementary Figure 1 and Supplementary Table 1. The voucher specimens were deposited at the State Key Laboratory of Quality Research in Chinese Medicine, Macau University of Science and Technology.

### Measurement of major components of ZiQin and KuQin by UHPLC-Q-TOF-MS analysis

According to the literature (Qiao et al., 2016), 10 mg of methanol extract powder was dissolved in 70% methanol (10 mL) with 30 min ultrasonication (40 kHz, 300 W). The sample was kept at room temperature for 5 min, and the supernatant was filtered through a 0.22 μm microporous membrane before use. An aliquot of 1 μL was injected into instrument for analysis.

An Agilent 6545 Accurate-Mass Q-TOF spectrometer coupled to an Agilent 1290 Infinity Binary LC system (UHPLC, Santa Clara, CA, United States) was employed. Samples were separated on an Agilent Eclipse Plus C18 column (1.8 μm, 2.1 mm ID × 150 mm). Mobile phase (A) was water containing formic acid (0.1%, v/v), and mobile phase (B) was acetonitrile and methanol (3:1, v/v). A gradient elution program was used as follows: 0–3 min, 80% A; 3–16 min, 80–64% A; 16–17 min, 64–40% A; 17–23 min, 40–20% A. The flow rate was 0.35 mL/min, and the column temperature was maintained at 55°C. The mass spectrometer was operated in the negative ESI mode. The parameters were as follows: Gas Temp: 325°C; Gas Flow:11 L/min; Sheath Gas Temp: 350°C; Sheath Gas Flow: 12 L/min; scan range: m/z 50–1700.

The file format of the raw data was converted by Abf (Analysis Base File) Converter software, and then peak extraction, peak alignment, and normalization were processed by MS-DIAL (Tsugawa et al., 2015) software. SIMCA 17 software was utilized for metabolomics data analysis.

### Active compound database construction for *Scutellariae radix*

The ingredients of *Scutellariae radix* were collected through the LC-MS identification (Table 1) and ETCM (Xu et al., 2019), TCMSP (Ru et al., 2014), and TCMID (Huang et al., 2018) database. The structures of the active compounds of *Scutellariae radix* were accustomed according to the PubChem database. The properties of absorption, distribution, metabolism, and excretion (ADME) were considered as important indicators for the effectiveness of drug candidates in modern drug discovery. The structures of the active ingredients from *Scutellariae radix* were then imported into “ADMET Descriptors” module in Discovery Studio 2021 (Dassault Systèmes BIOVIA, San Diego, CA) in SDF file format for the ADME prediction. Then these structures were put into the SwissADME (Daina et al., 2017) database for drug-like screening study.

**TABLE 1:** Identification of compounds from *Scutellariae radix* extract by UHPLC-Q-TOF-MS.

No.	t_*R*_(min)	[M-H]^–^ measured	(m/z) predicted	Δ(ppm)	MS/MS	Formula	Identification
1	3.69	303.0507	303.0510	−0.99	125.0244, 149.0242, 177.0183, 259.0613, 285.0396	C_15_H_12_O_7_	(2R,3R)-3,5,7,2′,6′-Pentahydroxyflavanone
2	5.33	547.1450	547.1457	−1.28	337.0721, 367.0821, 427.1012, 457.1129, 487.1224	C_26_H_28_O_13_	Chrysin 6-C-arabinoside 8-C-glucoside
3	6.13	623.1968	623.1981	−2.09	161.0238, 461.1636	C_29_H_36_O_15_	Acteoside
4	6.56	547.1448	547.1457	−1.64	337.0704, 367.0809, 427.1015, 457.1140	C_26_H_28_O_13_	Chrysin 6-C-β-D-glucoside-8-C-α-L-arabinoside
5	6.91	581.1857	581.1876	−3.27	299.0886, 329.1034, 461.1435	C_27_H_34_O_14_	2′,4′,6′-Trihydroxydihydrochalcone 3′-C-β-d glucoside-6′-O-β-D glucoside
6	7.16	547.1445	547.1457	−2.19	337.0687, 367.0807, 427.0995, 457.1117	C_26_H_28_O_13_	Chrysin 6-C-β-D-glucoside-8-C-β-L-arabinoside
7	8.41	285.0403	285.0405	−0.70	133.0291, 151.0040, 199.0357	C_15_H_10_O_6_	5,7,2′,6′-Tetrahydroxyflavone
8	10.22	345.0614	345.0616	−0.58	287.0164, 315.0139, 330.0369	C_17_H_14_O_8_	Viscidulin III
9	10.45	287.0560	287.0561	−0.35	125.0245, 161.0240	C_15_H_12_O_6_	2′,5,6′,7-Tetrahydroxyflavanone
10	10.91	285.0401	285.0405	−1.40	117.0345, 137.0240, 166.9946, 267.0338	C_15_H_10_O_6_	Scutellarein
11	11.11	445.0774	445.0776	−0.45	175.0245, 269.0448	C_21_H_18_O_11_	Baicalin
12	11.66	505.0975	505.0988	−2.57	299.0143, 314.0407, 329.0657	C_23_H_22_O_13_	Viscidulin II 2′-O-β-D-glucuronide
13	12.09	447.0926	447.0933	−1.57	243.0662, 271.0605	C_21_H_20_O_11_	Dihydrobaicalin
14	12.75	651.2276	651.2294	−2.76	160.0166, 175.0402, 193.0507, 475.1790	C_31_H_40_O_15_	Cistanoside D
15	12.98	445.0770	445.0776	−1.35	174.9556, 269.0450	C_21_H_18_O_11_	Norwogonin 8-O-β-D-glucuronide
16	14.04	429.0819	429.0827	−1.86	176.8982, 253.0511	C_21_H_18_O_10_	Chrysin 7-O-β-D-glucuronide
17	14.38	459.0931	459.0933	−0.44	113.0242, 175.0243, 283.0602	C_22_H_20_O_11_	Oroxylin A 7-O-β-D-glucuronide
18	14.97	445.0760	445.0776	−3.59	174.9557, 269.0450	C_21_H_18_O_11_	Baicalein 6-O-β-D-glucuronide
19	15.34	459.0928	459.0933	−1.09	113.0245, 268.0371, 283.0607	C_22_H_20_O_11_	Wogonoside
20	15.47	461.1079	461.1089	−2.17	175.0239, 270.0447, 285.0672	C_22_H_22_O_11_	(2S)-5,7-Dihydroxy-6-methoxyflavanone 7-O-β-D-glucuronide
21	16.28	489.1025	489.1038	−2.66	216.9330, 283.0246, 298.0480, 313.0706	C_23_H_22_O_12_	5,7-Dihydroxy-8,2′-Dimethoxyflavone 7-O-β-D-glucuronide
22	16.98	299.0555	299.0561	−2.01	136.9865, 256.0363, 284.0321	C_16_H_12_O_6_	4′-Hydroxywogonin
23	17.62	269.0455	269.0455	0	171.0458, 197.0602, 225.0567	C_15_H_10_O_5_	5,7,2′-Trihydroxyflavone
24	17.75	299.0554	299.0561	−2.34	136.9700, 256.0331, 284.0319	C_16_H_12_O_6_	Scutevulin
25	17.85	329.0661	329.0667	−1.82	109.9983, 165.9892, 314.0418	C_15_H_10_O_5_	5,7,6′-Trihydroxy-8,2′-dimethoxyflavone
26	17.97	299.0556	299.0561	−1.67	153.9866, 284.0327	C_16_H_12_O_6_	5,7,2′-Trihydroxy-6-methoxyflavone
27	18.05	269.0454	269.0455	−0.37	111.0081, 223.0478, 241.0499	C_15_H_10_O_5_	Baicalein
28	18.20	329.0662	329.0667	−1.52	299.0185, 314.0425	C_17_H_14_O_7_	5,8,2′-Trihydroxy-6,7-dimethoxyflavone
29	18.95	343.0816	343.0823	−2.04	285.0606, 298.8586, 313.1408	C_18_H_16_O_7_	Skullcapflavone
30	19.16	283.0609	283.0612	−1.06	184.0520, 211.0384, 239.0317, 268.0367	C_16_H_12_O_5_	Wogonin
31	19.23	253.0506	253.0506	0	107.0137, 143.0116, 209.1528	C_15_H_10_O_4_	Chrysin
32	19.28	373.0922	373.0929	−1.88	300.0257, 328.0128, 343.0433	C_19_H_18_O_8_	Skullcapflavone II (5,6′-Dihydroxy-6,7,8,2′-tetramethoxyflavone)
33	19.33	537.0810	537.0827	−3.17	245.0122, 391.0396	C_30_H_18_O_10_	8,8′-Bibaicalein
34	19.34	313.0712	313.0718	−1.92	283.0241, 298.0484	C_17_H_14_O_6_	5,8-Dihydroxy-6,7-dimethoxyflavone
35	19.48	283.0612	283.0612	0	239.0937, 268.0374	C_16_H_12_O_5_	Oroxylin A
36	19.89	343.0819	343.0823	−1.17	298.0075, 313.0370, 328.0593	C_18_H_16_O_7_	5,2′-Dihydroxy-6,7,8-trimethoxyflavone

### Target prediction of *Scutellariae radix* and COVID-19-associated target screening

The potential targets of the pharmacodynamic chemical compositions of *Scutellariae radix* were collected by SwissTargetPrediction (Daina et al., 2019) and ETCM databases. In order to better understand the mechanism of the disease, the COVID-19 related genes were collected from two resources (Yao et al., 2020). One was from database mining that the GeneCards^1^ were employed to obtain COVID-19-related genes. The other was from analysis of microarray data that the differential expressed genes (DEGs) were obtained by analyzing GSE147507 (Blanco-Melo et al., 2020), which was obtained from the GEO database^2^ via NetworkAnalyst online analysis with a threshold of adjust p-value ≤ 0.05 and | log2 FC| ≥ 1.0. A gene library of the anti-COVID-19 targets of *Scutellariae radix* was established by comparing and analyzing the targets shared by the COVID-19-associated targets and the predicted targets of *Scutellariae radix* (Liu et al., 2020).

### Bioinformatics analysis

To visualize and analyze the relationship between herbal medicine and disease, Cytoscape (version 3.7.2) (Shannon et al., 2003) was employed to construct the following three networks, “herb-compound-target-disease” network, the PPI network based on the importation of the gene symbols of the anti-COVID-19 targets of *Scutellariae radix* into the STRING (Szklarczyk et al., 2021) database, and target-signal pathway network.

The Metascape database^3^ (Zhou et al., 2019) is a web-based portal used for the analysis of GO (biological processes, molecular function, and cellular component) (Ashburner et al., 2000) and KEGG pathways (Kanehisa and Goto, 2000; Kanehisa et al., 2017). In this study, the gene symbols of the anti-COVID-19 targets of *Scutellariae radix* were imported into the Metascape platform, with Homo sapiens as the organism. The results of GO and KEGG pathway enrichment analysis were saved, and visualization was completed by the RStudio software.

### Molecular docking

The crystal structures of proteins were downloaded from RCSB Protein Data Bank. In each crystal structure, the ligand and protein were remained and other molecules like water were removed by Discovery Studio 2021. The position of the active pocket is defined by the ligand on the protein crystal structure. Every protein was prepared by “Prepare Protein” module in Discovery Studio 2021. The chemical structures of compounds were extracted from PubChem, and then optimized by using “Prepare Ligands” module. Finally, each compound was docked into the protein active site by “CDOCKER” module in Discovery Studio 2021 (Gagnon et al., 2016; Xu et al., 2021). CDOCKER is a precise semi-flexible molecular docking technology that has long been recognized by peer-reviewed journals (Gagnon et al., 2016).

### Cell lines and SARS-CoV-2 virus

HEK-293T cells, RAW264.7 cells, and the African green monkey epithelial (Vero E6) cells were supplied by American Type Culture Collection (ATCC, Rockville, MD, United States). HEK-293T-ACE2*^h^* cells were constructed by Sino Biological (Beijing, China). All the cell lines were cultured in Dulbecco Modified Eagle Medium (DMEM) supplemented with 10% fetal bovine serum and 1% Gibco penicillin-streptomycin-glutamine (Thermo Fisher Scientific, Waltham, United States) at 37°C in a humidified atmosphere of 5% CO_2_. Besides, 100 μg/mL hygromycin was used for the culture of HEK-293T-ACE2*^h^* cells. SARS-CoV-2 (Genebank accession no. MT123290.1) was clinical isolate from the First Affiliated Hospital of Guangzhou Medical University. The viral titer was determined by 50% tissue culture infective dose (TCID_50_) according to the cytopathic effect by use of Reed-Muench method. All the infection experiments of SARS-CoV-2 strain were performed in a biosafety level-3 laboratory.

### Cell viability assay

The MTT assay was utilized to examine the cytotoxic effects of compounds on HEK-293T-ACE2*^h^* cells, RAW264.7 cells, and Vero E6 cells. HEK-293T-ACE2*^h^* cells were seeded into 96-well plate at a density of 1 × 10^4^ cells per well and then treated with different concentrations of compounds for 4 h to measure the maximum non-toxic concentration (CC_0_) of compounds. RAW264.7 cells (1 × 10^4^ cells/well) or Vero E6 cells (5 × 10^4^ cells/well) were seeded in 96-well plates for 24 h culture at 37°C in 5% CO_2_ ambience. Next, the cells were incubated with extracts or compounds at various concentrations. After an incubation of RAW264.7 cells for 24 h and Vero E6 cells for 72 h, 10 μL MTT (5 mg/mL) was administered to each well and incubated for another 4 h. The supernatants were then removed, and the formed formazan crystals were dissolved in 100 μL DMSO. Finally, the absorbance value of each well was measured by a microplate reader at 570 nm. All data were obtained in triplicates and presented as means ± SD.

### Quantitative detection of NO, tumor necrosis factor-α, and interleukin (IL)-6

RAW264.7 cells were seeded in 24-well (1 × 10^5^/well) plates and were pretreated with different concentrations of extracts or compounds for 1 h. The cells were then stimulated by LPS (100 ng/mL) for 18 h. The supernatant was then collected to determine NO production by using Griess reagent (Promega, Madison, WI, United States) according to the manufacturer’s protocol. The levels of TNF-α and IL-6 in the supernatant were quantified by using the corresponding ELISA kit (Cayman, Washtenau County, MI, United States) according to the manufacturer’s instructions (Lio et al., 2020).

### Biolayer interferometry binding assay

An Octet system (Octet RED96, ForteBio, United States) was employed for biolayer interferometry (BLI) to determine the binding kinetics and affinity of compounds or extracts with the proteins of SARS-CoV-2 spike RBD and human ACE2 (Sino Biological, Beijing, China). Either the recombinant His-tag human ACE2 (25 μg/mL aqueous solution) or recombinant His-tag SARS-CoV-2 spike RBD (25 μg/mL aqueous solution) was immobilized onto the surface of nitrilotriacetic acid (Ni-NTA) biosensors (Fortebio, United States) that were dipped in a range of concentrations of compounds or extracts. Background signal was subtracted from all samples by using a reference biosensor loaded with either SARS-CoV-2 spike RBD or ACE2, which did not receive compounds or extracts under identical condition. The subtracted sensorgrams were then fitted to a 1:1 binding model to calculate the resulting equilibrium dissociation constant (K_D_) value for this interaction. All experiments were repeated at least three times and K_D_ values were presented with mean ± SD.

### Pseudovirus-based assay for the infection of HEK293T-ACE2^h^ cells

A SARS-CoV-2 spike pseudovirus was constructed according to our published protocol (Kong et al., 2021; Xu et al., 2022). This pseudovirus system contains both the SARS-CoV-2’s spike protein and the Firefly luciferase reporter gene, which was employed to quantify compounds’ capacity to inhibit virus’s entry into host cells by measuring the luciferase’s activity after infection of HEK-293T-ACE2^h^ cells. Briefly, 1 × 10^4^ cells in 100 μL were seeded in 96-well plates and cultured for 24 h. Cells were first incubated in compound-containing media for 2 h at 37°C. After disposal of the media, 100 μL mixture solution of pseudovirus and compound (consisted of 30 μL pseudovirus, 20 μL media, and 50 μL two-fold concentration of compounds) was then transferred to HEK-293T-ACE2^h^ cells for 2 h incubation. After disposal of the above culture media, the cells were treated with fresh media for another 48 h of incubation at 37°C. The luciferase luminescence value was then read at 578 nm by a microplate reader (SpectraMax iD5 Multi-Mode Microplate Reader, Molecular Devices, United States) by using Firefly Luciferase Reporter Gene Assay Kit (China). The luciferase luminescence value of HEK-293T-ACE2^h^ cells infected with the SARS-CoV-2 spike pseudovirus was set as 100%. Pseudo virus inhibition rate (%) = [1 - (Sample fluorescence values - Average fluorescence value of the background)/(Average fluorescence value of virus control - Average fluorescence value of the background)] × 100%. The IC_50_ was determined by using the GraphPad Prism 8.0 software (La Jolla, CA, United States).

### Plaque reduction assay

A pretreatment mode was used in plaque reduction assay to test the inhibitory effect of compounds on SARS-CoV-2’s entry into host cells. The 50 plaque-forming units (PFU) of SARS-CoV-2 strains (Genebank accession no. MT123290.1) were pretreated with various concentrations of tested compounds at room temperature for 2 h. Then, the Vero E6 cell monolayers grown in 12-well plates were infected with the above mixture solution of both virus and compounds for 2 h incubation at 37°C. After removal of the above culture solution, the cell monolayers were covered with agar overlay (containing concentration: 0.6% agar and 2% FBS). The plates were subsequently incubated for 48 h at 37°C with 5% CO_2_ ambience, and followed by fixation in 10% formalin for 30 min. Then, the agar overlays were removed, and the cell monolayers were stained with 1% crystal violet. Finally, the plaques were counted and photographed. All the plaque reduction assays were carried out in triplicate, and IC_50_ values were expressed with mean ± SD.

### Statistical analysis

The data were expressed as the mean ± standard deviation (SD). Either t-test or non-parametric test was used to compare two groups. Statistical significance was set at *p* < 0.05. Analysis and graphing were performed by using GraphPad Prism 8.0 software (San Diego, CA, United States).

## Result

### Collection and screening of active compounds from *Scutellariae radix*

**FIGURE 2:**
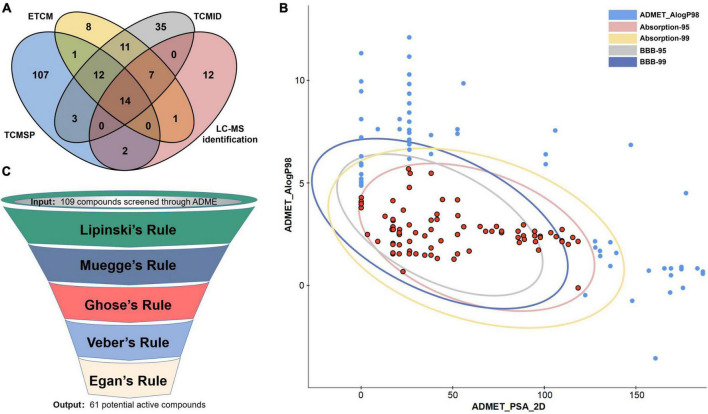
Screening of potential active compounds in *Scutellariae radix*. **(A)** Compounds in *Scutellariae radix* collected from various databases and LC-MS identification. **(B)** Bioavailability screening of compounds in *Scutellariae radix*. **(C)** Drug-likeness screening of compounds in *Scutellariae radix*.

As shown in Figure 2A, 213 compounds of *Scutellariae radix* were firstly collected based on ETCM (Xu et al., 2019), TCMSP (Ru et al., 2014), and TCMID (Huang et al., 2018) database and LC-MS identification (Table 1). 109 potential active ingredients were then filtered out from the components of *Scutellariae radix* according to the criterion of ADME (absorption, distribution, metabolism and excretion) provided by the Discovery Studio 2021 software (Figure 2B). This study also used five drug-likeness filters, including Lipinski (Pfizer) (Lipinski et al., 2001), Muegge (Bayer) (Muegge et al., 2001), Ghose (Amgen) (Ghose et al., 1999), Veber (GlaxoSmithKline) (Veber et al., 2002), and Egan (Pharmacia) (Egan et al., 2000). Finally, the ADME and drug-likeness screening yielded 61 potential active compounds (Figure 2C and Supplementary Table 2).

### Herb-compound-target-disease network construction and network topological analysis

A total of 445 targets were predicted through two chemical similarity-based target search methods (Daina et al., 2019; Xu et al., 2019) by using 61 potential active compounds of *Scutellariae radix* (Supplementary Tables 3, 4).

**FIGURE 3:**
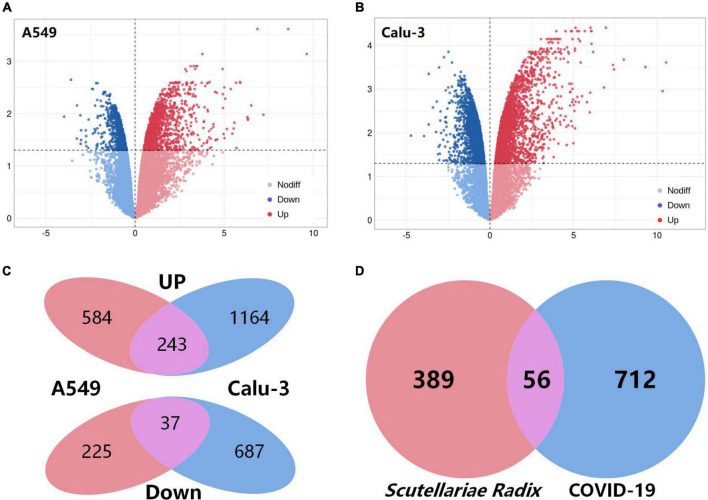
Acquisition of COVID-19-related targets and intersection targets. **(A,B)** Volcano plot of differentially expressed genes (comparison before and after SARS-CoV-2 infection) of A549 and calu-3 cell lines in GSE147507. Red: up-regulated; blue: down-regulated. **(C)** Venn diagram of common differential genes in A549 and calu-3 cell lines. **(D)** Venn diagram of potential targets of *Scutellariae radix* for the treatment of COVID-19.

COVID-19-associated genes were gathered from two different sources. First, the top 500 COVID-19-related genes were extracted from the GeneCards (Safran et al., 2010) database. Second, the transcriptomics analysis was used to investigate the genes associated with COVID-19 (Supplementary Table 5). The fold-changes between the two groups were depicted on the volcano plot (Figures 3A,B). As shown in Figure 3C, a total of 280 overlapping differentially expressed genes (DEGs) were screened out, including 243 up-regulated and 37 down-regulated DEGs (Supplementary Table 6). When the gene lists from the two sources were combined, 768 genes were thought to be potential targets for COVID-19 (Figure 3D).

**FIGURE 4:**
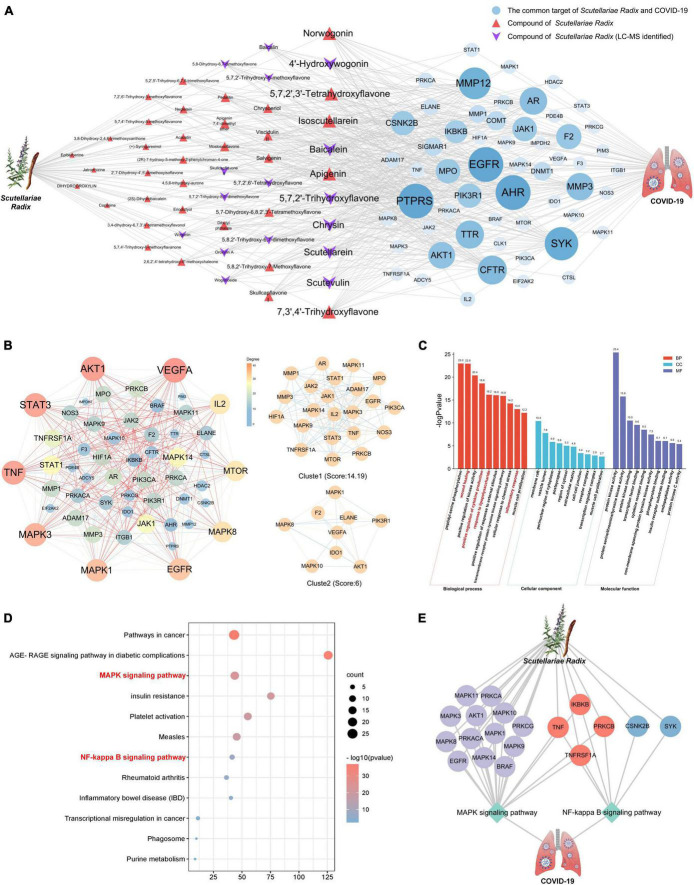
Network pharmacology analysis of *Scutellariae radix* against COVID-19. **(A)** Herb-Component-Target-Disease network diagram of *Scutellariae radix* against COVID-19. **(B)** The PPI network of targets of *Scutellariae radix* treating COVID-19. **(C)** GO enrichment analysis of common targets. **(D)** KEGG enrichment analysis of common targets. **(E)** Herb-Target-Pathway-Disease network diagram of *Scutellariae radix* against COVID-19. Light gray represents targets in the MAPK pathway. Blue represents targets in the NF-κB pathway. Red means the targets belong to both MAPK and NF-κB pathways.

The compound-target network and COVID-19 disease network were merged by Cytoscape software to construct a herb-compound-target-disease network that was composed of 108 nodes (1 herb, 50 compounds, 56 targets, and 1 disease) and 538 edges, as shown in Figure 4A. The composite network illuminated the relationship among the *Scutellariae radix*, candidate active compounds, potential therapeutic targets, and COVID-19. Based on the judgment criteria of previous network pharmacology research (Guo et al., 2022), it can be noticed that the top 10 active components in degree value (norwogonin, 4′-hydroxywogonin, 5,7,2′,3′-tetrahydroxyflavone, isoscutellarein, baicalein, apigenin, 5,7,2′-trihydroxyflavone, chrysin, scutellarein, scutevulin, and 7,3′,4′-trihydroxyflavone) are probably the main active ingredients of *Scutellariae radix* exerting anti-COVID-19 effect.

### PPI network, GO and KEGG analyses of the anti-COVID-19 targets of *Scutellariae radix*

To explore the possible mechanism of *Scutellariae radix* as treatment against COVID-19, 56 potential anti-COVID-19 targets of *Scutellariae radix* were imported to the STRING (Szklarczyk et al., 2021) database in order to construct a PPI network (Figure 4B). Further cluster analysis was done by using the MCODE module of Cytoscape (Shannon et al., 2003) to generate the highly connected sub-network. It can be noticed that the top 10 targets (VEGFA, AKT1, STAT3, TNF, MAPK3, MAPK1, EGFR, MAPK8, MTOR, and IL2) are potential hub genes of *Scutellariae radix* exerting anti-COVID-19 effect.

To further explore the various mechanisms of the anti-COVID-19 activity of *Scutellariae radix*, we performed a GO enrichment analysis (Ashburner et al., 2000) (biological processes, molecular function, and cellular component) of the 56 predicted targets. The top 10 enriched biological process terms, molecular function terms, and cellular component terms were determined (Figure 4C and Supplementary Table 7). The top five biological processes terms were peptidyl-serine phosphorylation, wound healing, positive regulation of kinase activity, positive regulation of cytokine production, and response to lipopolysaccharide. The top five molecular function terms were protein kinase activity, protein serine/threonine/tyrosine kinase activity, protein kinase binding, transcription factor binding, and cytokine receptor binding. The top five cellular component terms were membrane raft, vesicle lumen, perinuclear region of cytoplasm, postsynapse, and region of cytosol. Biological processes such as “wound healing, positive regulation of cytokine production, response to lipopolysaccharide, and inflammatory response” can be observed in the results of GO enrichment analysis. This result suggested that the anti-COVID-19 effect of *Scutellariae radix* was closely related to inflammation.

A KEGG pathway enrichment analysis (Kanehisa and Goto, 2000; Kanehisa et al., 2017) at *p* < 0.01 significance level was performed for the 56 potential anti-COVID-19 targets of *Scutellariae radix* (Figure 4D and Supplementary Table 8). The top ten significantly enriched KEGG pathways were Pathways in cancer (hsa05200), AGE-RAGE signaling pathway in diabetic complications (hsa04933), MAPK signaling pathway (hsa04010), Insulin resistance (hsa04931), Platelet activation (hsa04611), Measles (hsa05162), NF-kappa B signaling pathway (hsa04064), Rheumatoid arthritis (hsa05323), Inflammatory bowel disease (IBD) (hsa05321) and Transcriptional misregulation in cancer (hsa05202). Notably, MAPK and NF-κB signaling pathway are significant pathways regulating inflammatory cytokine storm. According to the results from GO and KEGG analyses, the potential mechanism of *Scutellariae radix* exerting anti-COVID-19 effect may be achieved by regulating MAPK and NF-κB signaling pathways by the active ingredients of *Scutellariae radix* (Figure 4E).

### Molecular docking predicted main active components of *Scutellariae radix* against COVID-19

**FIGURE 5:**
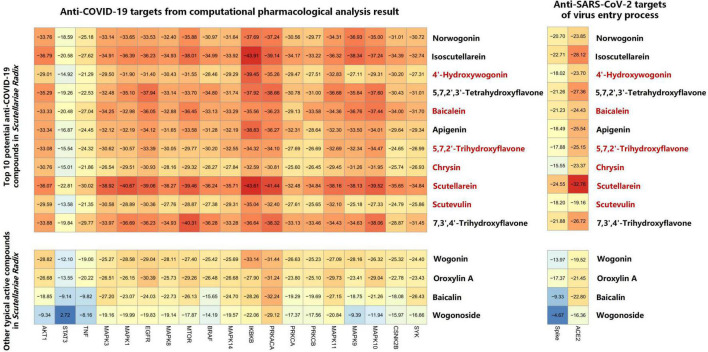
Heatmap of molecular docking analysis The top 10 potential active compounds that can be detected by LC-MS in *Scutellariae radix* were marked in red.

Specific parameter setting information for molecular docking was presented in Supplementary Table 9. According to the results of network pharmacology, the targets in the MAPK and NF-κB pathways, and the top 10 targets in the PPI network with degree value were considered as the main potential targets of *Scutellariae radix* against COVID-19. Two additional targets (SARS-CoV-2 spike protein, and human ACE2 receptor) closely related to the direct infection of host cells by SARS-CoV-2 have also been included in this study, due to the reported anti-infectious ability of *Scutellariae radix* in other bacterial or viral diseases. A precise semi-flexible molecular docking method of CDOCKER (Gagnon et al., 2016; Xu et al., 2021) was used to further investigate the main components of *Scutellariae radix* for treating COVID-19. The top 10 compounds in network pharmacology analysis and another four constituents (wogonin, oroxylin A, baicalin, and wogonoside) that were often considered to be the active ingredients of *Scutellariae radix*, were docked into the active centers of the aforementioned targets. According to the result of molecular docking (Figure 5), the top five constituents with high docking scores against COVID-19 were scutellarein, isoscutellarein, 7,3′,4′-trihydroxyflavone, 5,7,2′,3′-tetrahydroxyflavone, and baicalein.

Lower binding energy indicated higher stability. Based on the binding energy score (Figure 5), IKBKB was identified as a key target of *Scutellariae radix* for the treatment of COVID-19 through anti-inflammatory effects. IKBKB plays an essential role in the NF-κB signaling pathway which is a key regulatory target in the activation process of NF-κB (Cardinez et al., 2018). As displayed in Supplementary Figure 2, scutellarein formed four hydrogen bonds with Lys44, Glu97, Cys99, and Asp166 in IKBKB. In addition, two hydrogen bonds were formed between baicalein and IKBKB (Supplementary Figure 2). The binding energies of scutellarein and baicalein with IKBKB were - 43.61 and - 35.56 kJ/mol, respectively. The results indicated that the components could bind to the active sites of the targets. The surface envelops spike protein of SARS-CoV-2 is essential for its infection of host cells. The initial attachment of the SARS-CoV-2 to the host cell is initiated by interactions between the spike RBD and human receptor ACE2. According to the relevant literature (Lan et al., 2020; Teli et al., 2021), the key amino acid residues for spike to bind to ACE2 are Tyr449, Gln493, Gln498, Thr500, Asn501, Gly502, and Tyr505. As shown in Supplementary Figure 2C, scutellarein formed three hydrogen bonds with Arg403, Tyr453, and Ser494 in spike protein. Supplementary Figure 2D showed that baicalein could form stable hydrogen bond with spike protein Gln498, a key amino acid residue in the binding of spike protein to human ACE2 protein. In addition, baicalein also formed hydrogen bonds with spike protein Ser494 and Gly496. According to molecular docking results, scutellarein and baicalein had the potential to prevent SARS-CoV-2 entry into host cells by blocking viral spike RBD.

### Chemical analysis of *Scutellariae radix* by UHPLC-Q-TOF-MS

**FIGURE 6:**
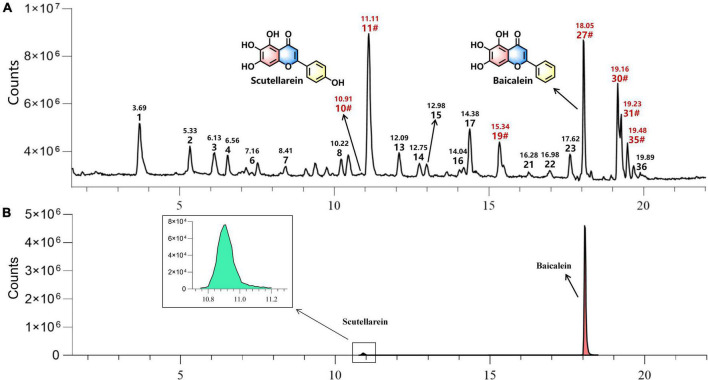
The representative UHPLC-Q-TOF-MS chromatograms of extract of *Scutellariae radix* in negative mode The label of “#” indicated that compounds were confirmed by reference standards with indicated retention time. **(A)** Typical total ion chromatogram (TIC) of *Scutellariae radix* extract. **(B)** Typical extracted ion chromatogram (EIC) of scutellarein and baicalein in the extract of *Scutellariae radix*.

A total of 36 compounds were identified from two commercial products specification (ZiQin and KuQin) of *Scutellariae radix* by UHPLC-Q-TOF-MS analysis (Figure 6, Supplementary Figure 3, and Table 1). Only scutellarein and baicalein were found in the extract of *Scutellariae radix* out of the top 5 compounds revealed by molecular docking study. The other three constituents (isoscutellarein, 7,3′,4′-trihydroxyflavone and 5,7,2′,3′-tetrahydroxyflavone) were too trace to be detected from *Scutellariae radix* under our tested UHPLC-Q-TOF-MS condition. Therefore, scutellarein and baicalein were selected for subsequent biological verification by bioassays.

### Anti-inflammatory and anti-infectious potential of *Scutellariae radix*

**FIGURE 7:**
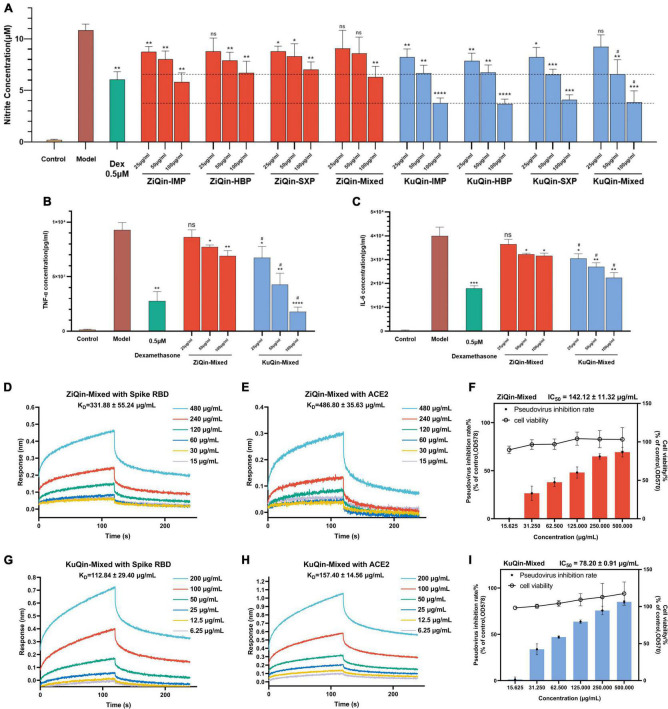
Anti-inflammatory and anti-infectious potential of *Scutellariae radix*. **(A–C)** Effects of the extracts of ZiQin and KuQin on the levels of NO, TNF-α, and IL-6 in LPS-stimulated RAW264.7 cells. The cells were plated in 24-well plates and incubated for 18 h, pretreated with different concentrations of the extracts for 1 h and then stimulated with LPS (100 ng/ml) for 18 h. The concentrations of NO, TNF-α, and IL-6 in culture medium were quantified by Griess Reagent System or ELISA assay (Dex: Dexamethasone). **(D,E,G,H)** Binding curves of ZiQin-Mixed and KuQin-Mixed with SARS-CoV-2 spike RBD and human ACE2 proteins by BLI binding kinetics assay. **(F,I)** ZiQin-Mixed, and KuQin-Mixed inhibit SARS-CoV-2 pseudovirus into HEK-293T-ACE2^h^ cells under non-toxic conditions to host cells (HBP: Hebei province; IMP: Inner Monglia Autonomous Region; SXP: Shanxi province; Mixed: All samples in the same category were mixed in equal proportions.) All the experiments were repeated at least three times. *p < 0.05, **p < 0.01, ***p < 0.001, ****p < 0.0001, compared with model group; ^#^*p* < 0.05, compared with ZiQin under the same sample concentration.

LPS-stimulated mouse macrophage RAW264.7 cells have been wildly used as an inflammatory cell model to evaluate anti-inflammatory activity of drugs (Huang et al., 2013). Therefore, this study utilized this inflammation model to simulate and explore the anti-inflammatory effect of the extracts of *Scutellariae radix* in COVID-19. The cytotoxicity of the extracts of ZiQin and KuQin in RAW264.7 was firstly examined by the MTT assay (Supplementary Figure 4). The extracts of both ZiQin and KuQin showed no significant cytotoxicity on RAW264.7 cells under the concentration of 400 μg/ml. There was no significant difference in the inhibitory effect of ZiQin extracts from three different origins (Inner Monglia autonomous region, Hebei province and Shanxi province) on the NO release in the RAW264.7 inflammatory cell model, and so did KuQin (Figure 7A). Dose-dependent inhibition of NO release was observed for both ZiQin and KuQin extracts. ELISA assay was also used to determine the protein expression levels of TNF-α and IL-6, two inflammation cytokines in RAW264.7 inflammatory cell model. As the results, both ZiQin and KuQin extracts dose-dependently suppressed the release of TNF-α and IL-6 (Figures 7A–C). In addition, the inhibitory effects of KuQin extract on the release of NO, TNF-α and IL-6 were more potent than that of ZiQin extract (*p* < 0.05) at the concentration of 50 μg/ml and 100 μg/ml (Figures 7B,C). These results suggested that *Scutellariae radix* has anti-inflammatory potential, and the anti-inflammatory effect of KuQin extract was more potent than that of ZiQin extract.

SARS-CoV-2 enters the host cells by recognizing ACE2 receptor of human cells. Biolayer interference (BLI) binding analysis was conducted to further evaluate the binding ability of extracts of *Scutellariae radix* with both the spike RBD and human ACE2 proteins, which could affect the recognition of SARS-CoV-2 spike RBD to human ACE2 receptor. As shown in Figures 7D,E,G,H, it was found that KuQin extract binds to the spike RBD with an affinity (K_D_ value) of 112.84 ± 29.40 μg/mL, stronger than that (331.88 ± 55.24 μg/mL) of ZiQin extract. KuQin extract binds to human ACE2 protein with a K_D_ value of 157.40 ± 14.56 μg/mL, while ZiQin gives a K_D_ value of 486.80 ± 35.63 μg/mL. In general, the binding affinity of KuQin with both SARS-CoV-2 spike RBD and human ACE2 proteins was significantly higher than that of ZiQin (*p* < 0.01). KuQin and ZiQin were further evaluated for their potential to inhibit viral entry of a SARS-CoV-2 spike pseudovirus bearing Firefly luciferase reporter into HEK-293T-ACE2^h^ host cells. As a consequence, the ability of SARS-CoV-2 spike pseudovirus entering into host cells was significantly reduced by the extracts of both KuQin and ZiQin, where IC_50_ value (78.20 ± 0.91 μg/mL) of KuQin was approximately half that (142.12 ± 11.32 μg/mL) of ZiQin (*p* < 0.0001) (Figures 7F,I), indicating a stronger antiviral activity of KuQin than ZiQin. Taken together, these results suggested that *Scutellariae radix* possesses both anti-inflammatory and anti-infectious potential. Furthermore, KuQin had superior anti-inflammatory and anti-SARS-CoV-2 activities to ZiQin.

### Validation of main active components of *Scutellariae radix* for anti-inflammatory and anti-infectious effects

**FIGURE 8:**
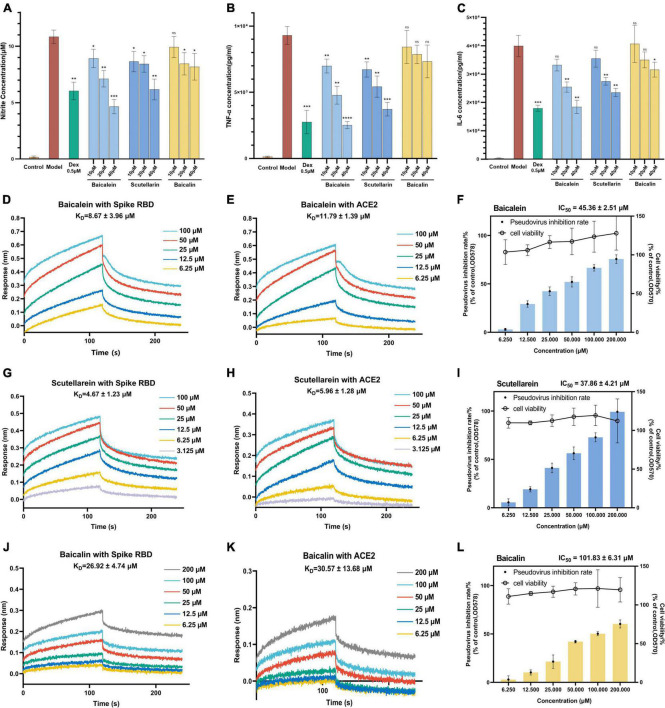
*In vitro* experiments confirmed that baicalein and scutellarein are main active anti-COVID-19 components in KuQin. **(A–C)** Effects of baicalein, scutellarein, and baicalin on the levels of NO, TNF-α, and IL-6 in LPS-stimulated RAW264.7 cell. The concentrations of NO, TNF-α, and IL-6 in culture medium were quantified by Griess Reagent System or ELISA assay. **(D,E,G,H,J,K)** Binding curves of baicalein, scutellarein and baicalin with SARS-CoV-2 spike RBD and human ACE2 receptor. **(F,I,L)** Baicalein, scutellarein and baicalin inhibited the entrance of SARS-CoV-2 pseudovirus into host cells. All the experiments were repeated three times. *p < 0.05, **p < 0.01, ***p < 0.001, ****p < 0.0001, compared with model group.

The effects of baicalein and scutellarein on the levels of NO, TNF-α, and IL-6 in LPS stimulated RAW264.7 cells were subsequently evaluated by using the same above bioassays. Baicalin, as the only chemical marker for quality control of *Scutellariae radix* in Chinese Pharmacopoeia, was also evaluated for its activity. As shown in Figures 8A-C, it was found that baicalein and scutellarein demonstrated more potent inhibitory effects on the production of NO, TNF-α, and IL-6 than baicalin. Based on these results, it was considered that bacalein and scutellarein were active components with anti-inflammatory potential in *Scutellariae radix*.

As shown in Figures 8D,E,G,H,J,K, BLI binding assay revealed that baicalein, scutellarein and baicalin dose-dependently bind to both SARS-CoV-2 spike RBD and ACE2 proteins. The binding affinity of baicalein and scutellarein were 2.6 ∼ 5.7 folds higher than that of baicalin with both proteins. Baicalein and scutellarein might more effectively prevent SARS-CoV-2 from binding to the host’s ACE2 receptor by dual recognizing both spike RBD and human ACE2 proteins than baicalin. As shown in Figures 8F,I,L, baicalein, scutellarein and baicalin exhibited antiviral entry effect on SARS-CoV-2 spike pseudoviruses into host cells with IC_50_ values of 45.36, 37.86, and 101.83 μM, respectively. Based on both strong anti-inflamamatory and anti-infectious potency, baicalein was selected for subsequent antiviral validation against SARS-CoV-2, and baicalin (the glycoside of baicalein) was also used for comparison.

### Antiviral effect of baicalein against SARS-CoV-2 by plaque formation assay

**FIGURE 9:**
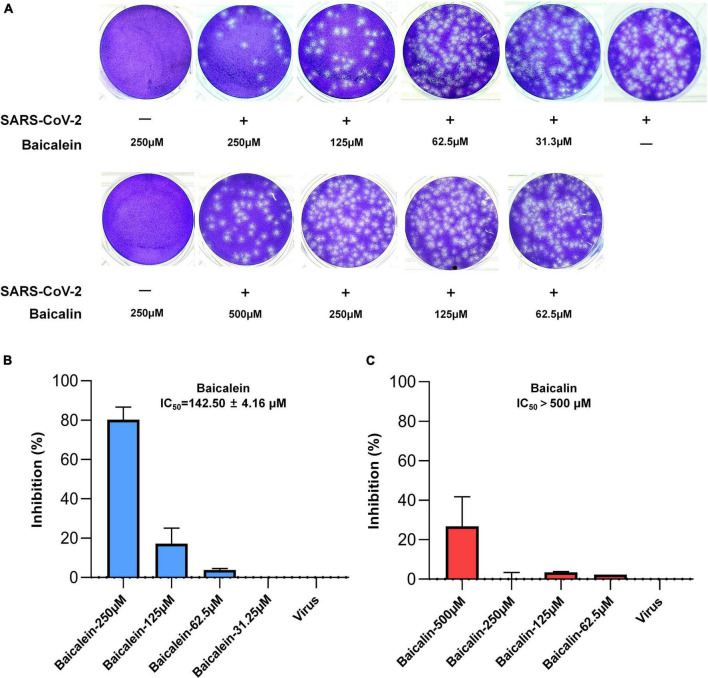
The antiviral effects of baicalein and baicalin against SARS-CoV-2 virus by plaque reduction assay. **(A)** The photographs of plaque reduction assay treated with different concentrations of compounds. Inhibitory activities of **(B)** baicalein and **(C)** baicalin against SARS-CoV-2.

In order to confirm the above antiviral effect of baicalein against SARS-CoV-2, plaque formation assay was further carried out in a biosafety level 3 facility. Baicalein and baicalin were not cytotoxic on Vero E6 cells at a tested concentration up to 250 and 500 μM, respectively. In a pretreatment mode, baicalein showed a dose-dependent inhibitory effect on SARS-CoV-2’s entry into Vero E6 host cells with an IC_50_ value of 142.50 μM by plaque formation assay, while baicalin displayed extremely weak antiviral activity even in the treatment concentration of 500 μM (Figure 9). The antiviral entry effect of baicalein against SARS-CoV-2 further confirmed the result from pseudovirus model.

### KuQin’s chemical basis for superior anti-COVID-19 potential to ZiQin

**FIGURE 10:**
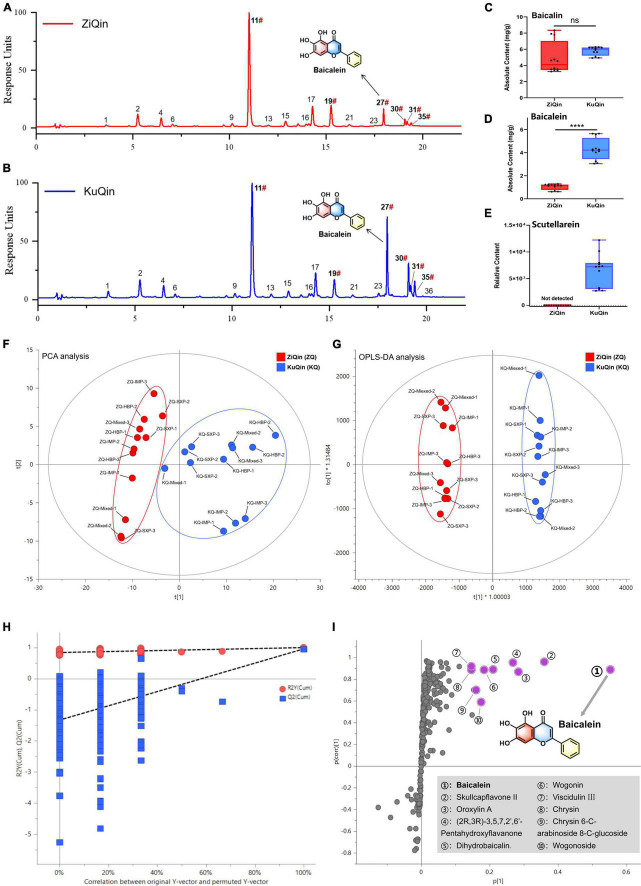
Chemical comparison between ZiQin and KuQin. **(A,B)** UHPLC-UV chromatograms of ZiQin and KuQin extract under UV wavelength of 270 nm. The label of “#” indicated that compounds were confirmed by reference standards. **(C,D)** Quantitative analysis of baicalin and baicalein in ZiQin (red) and KuQin (blue) extracts by UHPLC-UV with an external standard method, respectively. **(E)** The relative content of scutellarein under MS detection. **(F,G)** The PCA analysis and OPLS-DA analysis between ZiQin and KuQin. **(H)** Permutation test (*n* = 200) demonstrated no overfitting in the OPLS-DA model. **(I)** S-plot diagram from the OPLS-DA model between ZiQin and KuQin. The points highlighted in purple implied the compounds with significantly higher content in KuQin than those in ZiQin (HBP, Hebei province; IMP, Inner Monglia Autonomous Region; SXP, Shanxi province; Mixed, All samples in the same category were mixed in equal proportions).

It was found that KuQin outperforms ZiQin in both anti-inflammatory and anti-infectious activity against SARS-CoV-2 based on the above results. The chemical difference of KuQin and ZiQin was thus analyzed by UHPLC-UV instrument coupled with an ESI-TOF detector (Figures 10A–E). In order to avoid the influence of sample origin difference on the accuracy of this study, we have collected 3 batches of samples from three provinces (1 batch/province) for the chemical analysis of both ZiQin and KuQin. Figures 10A,B showed the typical UHPLC-UV chromatograms of KuQin and ZiQin. KuQin showed a very similar chromatogram to that of ZiQin except for a remarkably higher content of baicalein (peak No. 27). Quantitation was performed by external standard method. The content of baicalin in KuQin was comparable to that in ZiQin (Figure 10C and Supplementary Table 10), but the content of baicalein in KuQin was 4.1-fold higher than that in ZiQin (Figure 10D and Supplementary Table 10). However, scutellarein was too trace to be detected at UV wavelength of 270 nm under our tested condition. This constituent can be detected by MS detector only in KuQin, but not in ZiQin (Figure 10E).

Principal Component Analysis (PCA) was then carried out to district the metabolic profiles between KuQin and ZiQin. According to the PCA score graph (Figure 10F), it can be seen intuitively that ZiQin and KuQin samples were divided into two categories, suggesting that regional differences did not have significant impact on the chemical composition of the two commercial products specification. OPLS-DA model was also used to further explore the specific chemical differences between ZiQin and KuQin. The established OPLS-DA model presented satisfied explanation and prediction according to the cross-validations analysis (Figure 10G), where R^2^X_(cum)_ = 0.906, R^2^Y_(cum)_ = 0.996 and Q^2^_(cum)_ = 0.954. Permutation test with 200 permutations also showed that Q^2^ and R^2^ value were lower than the original points, which suggested that the established model was deemed to be successful (Figure 10H). According to these two criteria, baicalein can be considered as the most significantly differential chemical marker of KuQin compared to ZiQin (Figure 10I).

## Discussion

Globally, since the outbreak of COVID-19 in December 2019, WHO has reported 572 million confirmed cases of COVID-19, including 6.39 million deaths. Until now, the prevention and treatment of COVID-19 remain a major global challenge. China mainly treats mild and ordinary patients with traditional Chinese medicine (TCM) to improve clinical symptoms such as fever, cough, anorexia, and fatigue, as well as to reduce the development of severe diseases. Through data mining of the official COVID-19 treatment guidelines of 34 provinces in China, we found that *Scutellariae radix* is extensively used in the treatment of non-severe patients. According to the theory of TCM, *Scutellariae radix* is divided into two specifications: ZiQin and KuQin. Ancient books of TCM have recorded that KuQin is better than ZiQin in the treatment of pneumonia, but the opposite in the treatment of enteritis. Essentially, COVID-19 is a pneumonia caused by SARS-CoV-2 infection. It has not yet been reported whether the aforementioned TCM theories are applicable in the treatment of COVID-19, and what the mechanism is. However, due to the complex chemical components of *Scutellariae radix*, it remains difficult to elucidate its potential active compounds and precise pharmacological mechanisms in treating COVID-19.

**FIGURE 11:**
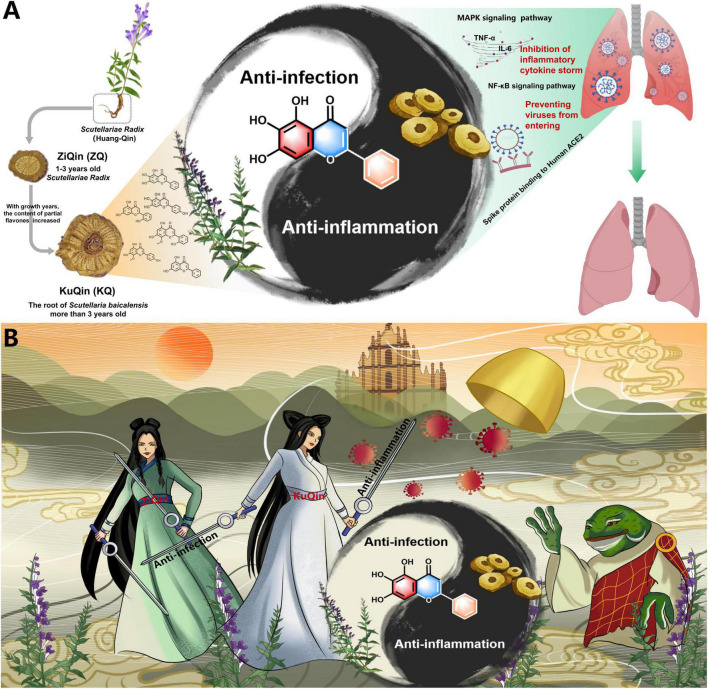
Summary of the mechanism of *Scutellariae radix* for the treatment of COVID-19. **(A)** An academic summary of the mechanism. **(B)** An artistic summary of the mechanism. In Chinese folklore, a white snake and a green snake become immortal through cultivation and offer generous healing to the poor in the form of beautiful ladies. The white snake’s magic power is stronger than the green snake because white snake has a thousand years of magic power and green snake has only practiced for five hundred years. Their enemy is a toad-turned-demon that has unleashed a plague in the world.

The bioactive components and therapeutic mechanisms of *Scutellariae radix* were summarized in Figure 11 for the treatment of COVID-19. In this research, network pharmacology and molecular docking analysis revealed that *Scutellariae radix* primarily regulated the MAPK and NF-κB signaling pathways *via* active components such as scutellarein and baicalein, and blocked viral spike protein binding to ACE2 receptors to treat COVID-19. *In vitro* bioassays showed that scutellarein and baicalein were proved to possess more potent anti-inflammatory and anti-infectious activities than baicalin, the dominant principle with the highest content in *Scutellariae radix*. The biological activities of scutellarein and baicalein were comparable in the treatment of COVID-19. With respect to the relative content in *Scutellariae radix*, baicalein is one of the main constituents, while scutellarein is just a trace component. Moreover, baicalein was confirmed by plaque formation assay to inhibit SARS-CoV-2’s entry into Vero E6 cells with an IC_50_ value of 142.50 μM. In summary, baicalein was considered to be the most important active component of *Scutellariae radix* for the treatment of COVID-19 by integrative analysis.

The *in vitro* bioassays demonstrated that KuQin outperforms ZiQin both in the anti-inflammatory and anti-infectious activities. Using non-targeted metabolomics technology, we discovered that the content of active aglycones in KuQin was significantly higher than that of ZiQin. It is worth mentioning that baicalein’s VIP value (the value of variable important in projection) exceeded 1.58 times that of the second-ranked compound. This result suggested that baicalein was the compound with the most significant variation in KuQin compared to ZiQin. Quantitative analysis showed that the content of baicalein in KuQin was 4.1-fold higher than that in ZiQin. In summary, we considered that the significant increase of baicalein in KuQin is the main reason for the superiority of KuQin over ZiQin in the treatment of COVID-19. Based on the above quality evaluation of KuQin and ZiQin, this study also provided a scientific support for the traditional theory of TCM that KuQin was good at cooling down the heat in the lung. These findings suggested that KuQin could be used as a dietary supplement for COVID-19 patients.

In Chinese Pharmacopoeia (2020 version), baicalin was used as the only chemical marker for the quality control of *Scutellariae radix*. It was suggested that baicalein could be employed as an additional bioactive marker for the quality assurance of *Scutellariae radix* based on this study.

## Conclusion

In summary, our study explored the potential bioactive components, underlying mechanism and quality of the widely used dietary supplement, *Scutellariae radix* for the treatment of COVID-19 by using network pharmacology, molecular docking, LC-MS analysis and *in vitro* biological evaluations. This investigation provided both theoretical and experimental supports for the treatment of COVID-19 with *Scutellariae radix*. Based on the findings of this study, KuQin was better than ZiQin for the prescriptions of TCMs treating COVID-19 due to its superior anti-inflammatory and anti-infectious effects to ZiQin. Baicalein was the most effective component with both anti-inflammatory and antiviral efficacy in *Scutellariae radix*. Therefore, baicalein was suggested to be used as an additional bioactive marker for the quality assurance of *Scutellariae radix* based on this study.

## Data availability statement

The datasets presented in this study can be found in online repositories. The names of the repository/repositories and accession number(s) can be found in the article/Supplementary material.

## Author contributions

L-PB and Z-HJ conceived and designed the whole study. Z-FY and RL designed the antiviral bioassay with SARS-CoV-2. JL performed the network pharmacology analysis, molecular docking, and LC-MS analysis and anti-inflammatory evaluations. JM, HJ, and RL performed all the antiviral experiments. LF and TX assisted the completion of some biological experiments. JL and JM carried out statistical analysis, chart drawing, and drafted the manuscript. L-PB and Z-HJ revised the manuscript. G-YZ and WZ gave comments on the revision of the manuscript. JG assisted the quantitative analysis of baicalein and baicalin in *Scutellariae radix*. L-PB and Z-HJ obtained the funding and supervised the whole study. All the authors approved the final version of the manuscript.

## Footnotes

1
https://www.genecards.org/

2
https://www.ncbi.nlm.nih.gov/

3
http://metascape.org/

## References

[B1] Ashburner M. Ball C. A. Blake J. A. Botstein D. Butler H. Cherry J. M. (2000). Gene ontology: tool for the unification of biology. *Nat. Genet.* 25 25–29. 10.1038/75556 10802651 PMC3037419

[B2] Blanco-Melo D. Nilsson-Payant B. E. Liu W.-C. Uhl S. Hoagland D. Møller R. (2020). Imbalanced host response to SARS-CoV-2 drives development of COVID-19. *Cell* 181 1036–1045. 10.1016/j.cell.2020.04.026 32416070 PMC7227586

[B3] Cao X. You G. Li H. Li D. Wang M. Ren X. (2019). Comparative investigation for rotten xylem (kuqin) and strip types (tiaoqin) of *Scutellaria baicalensis* Georgi based on fingerprinting and chemical pattern recognition. *Molecules* 24:2431. 10.3390/molecules24132431 31269661 PMC6651509

[B4] Cardinez C. Miraghazadeh B. Tanita K. da Silva E. Hoshino A. Okada S. (2018). Gain-of-function IKBKB mutation causes human combined immune deficiency. *J. Exp. Med.* 215 2715–2724. 10.1084/jem.20180639 30337470 PMC6219745

[B5] Daina A. Michielin O. Zoete V. (2017). SwissADME: a free web tool to evaluate pharmacokinetics, drug-likeness and medicinal chemistry friendliness of small molecules. *Sci. Rep.* 7 1–13. 10.1038/srep42717 28256516 PMC5335600

[B6] Daina A. Michielin O. Zoete V. (2019). SwissTargetPrediction: updated data and new features for efficient prediction of protein targets of small molecules. *Nucleic Acids Res.* 47 W357–W364. 10.1093/nar/gkz382 31106366 PMC6602486

[B7] Delerue T. Barroso M. F. Dias-Teixeira M. Figueiredo-González M. Delerue-Matos C. Grosso C. (2021). Interactions between *Ginkgo biloba* L. and *Scutellaria baicalensis* Georgi in multicomponent mixtures towards cholinesterase inhibition and ROS scavenging. *Food Res. Int.* 140:109857. 10.1016/j.foodres.2020.109857 33648175

[B8] Deng Y. Ren H. Ye X. Xia L. Liu M. Liu Y. (2020). Integrated phytochemical analysis based on UPLC-Q-TOF-MS/MS, network pharmacology, and experiment verification to explore the potential mechanism of platycodon grandiflorum for chronic bronchitis. *Front. Pharmacol.* 11:564131. 10.3389/fphar.2020.564131 33013400 PMC7506058

[B9] Dinda B. Dinda S. DasSharma S. Banik R. Chakraborty A. Dinda M. (2017). Therapeutic potentials of baicalin and its aglycone, baicalein against inflammatory disorders. *Eur. J. Med. Chem.* 131 68–80. 10.1016/j.ejmech.2017.03.004 28288320

[B10] Egan W. J. Merz K. M. Baldwin J. J. (2000). Prediction of drug absorption using multivariate statistics. *J. Med. Chem.* 43 3867–3877. 10.1021/jm000292e 11052792

[B11] Gagnon J. K. Law S. M. Brooks C. L. III (2016). Flexible CDOCKER: development and application of a pseudo-explicit structure-based docking method within CHARMM. *J. Comput. Chem.* 37 753–762. 10.1002/jcc.24259 26691274 PMC4776757

[B12] Gao J. Ding Y. Wang Y. Liang P. Zhang L. Liu R. (2021). Oroxylin A is a severe acute respiratory syndrome coronavirus 2-spiked pseudotyped virus blocker obtained from Radix Scutellariae using angiotensin-converting enzyme II/cell membrane chromatography. *Phytother. Res.* 35 3194–3204. 10.1002/ptr.7030 33587321 PMC8013958

[B13] Ghose A. K. Viswanadhan V. N. Wendoloski J. J. (1999). A knowledge-based approach in designing combinatorial or medicinal chemistry libraries for drug discovery. 1. A qualitative and quantitative characterization of known drug databases. *J. Comb. Chem.* 1 55–68. 10.1021/cc9800071 10746014

[B14] Guo B. Zhao C. Zhang C. Xiao Y. Yan G. Liu L. (2022). Elucidation of the anti-inflammatory mechanism of Er Miao San by integrative approach of network pharmacology and experimental verification. *Pharmacol. Res.* 175:106000. 10.1016/j.phrs.2021.106000 34838694

[B15] Huang J. Qin Y. Liu B. Li G. Ouyang L. Wang J. (2013). In silico analysis and experimental validation of molecular mechanisms of salvianolic acid A-inhibited LPS-stimulated inflammation, in RAW264.7 macrophages. *Cell Prolif.* 46 595–605. 10.1111/cpr.12056 24033467 PMC6496881

[B16] Huang L. Xie D. Yu Y. Liu H. Shi Y. Shi T. (2018). TCMID 2.0: a comprehensive resource for TCM. *Nucleic Acids Res.* 46 D1117–D1120. 10.1093/nar/gkx1028 29106634 PMC5753259

[B17] Kanehisa M. Goto S. (2000). KEGG: kyoto encyclopedia of genes and genomes. *Nucleic Acids Res.* 28 27–30. 10.1093/nar/28.1.27 10592173 PMC102409

[B18] Kanehisa M. Furumichi M. Tanabe M. Sato Y. Morishima K. (2017). KEGG: new perspectives on genomes, pathways, diseases and drugs. *Nucleic Acids Res.* 45 D353–D361. 10.1093/nar/gkw1092 27899662 PMC5210567

[B19] Kong L. Meng J. Tian W. Liu J. Hu X. Jiang Z.-H. (2021). I2-catalyzed carbonylation of α-methylene ketones to synthesize 1, 2-diaryl diketones and antiviral quinoxalines in one pot. *ACS Omega* 7 1380–1394. 10.1021/acsomega.1c06017 35036799 PMC8757360

[B20] Lan J. Ge J. Yu J. Shan S. Zhou H. Fan S. (2020). Structure of the SARS-CoV-2 spike receptor-binding domain bound to the ACE2 receptor. *Nature* 581 215–220. 10.1038/s41586-020-2180-5 32225176

[B21] Li L. Wu Y. Wang J. Yan H. Lu J. Wang Y. (2021). Potential treatment of COVID-19 with traditional Chinese medicine: what herbs can help win the battle with SARS-CoV-2? *Engineering*. 10.1016/j.eng.2021.08.020 [Epub ahead of print]. 34729244 PMC8552808

[B22] Li Z.-T. Zhang F.-X. Fan C.-L. Ye M.-N. Chen W.-W. Yao Z.-H. (2021). Discovery of potential Q-marker of traditional Chinese medicine based on plant metabolomics and network pharmacology: periplocae cortex as an example. *Phytomedicine* 85:153535. 10.1016/j.phymed.2021.153535 33819766

[B23] Liao H. Ye J. Gao L. Liu Y. (2021). The main bioactive compounds of *Scutellaria baicalensis* Georgi. for alleviation of inflammatory cytokines: a comprehensive review. *Biomed. Pharmacother.* 133:110917. 10.1016/j.biopha.2020.110917 33217688

[B24] Lio C.-K. Luo J.-F. Shen X.-Y. Dai Y. Machado J. Xie Y. (2020). Nardosinanone N suppresses LPS-induced macrophage activation by modulating the Nrf2 pathway and mPGES-1. *Biochem. Pharmacol.* 173:113639. 10.1016/j.bcp.2019.113639 31536727

[B25] Lipinski C. A. Lombardo F. Dominy B. W. Feeney P. J. (2001). Experimental and computational approaches to estimate solubility and permeability in drug discovery and development settings 1. *Adv. Drug Delivery Rev.* 46 3–26. 10.1016/S0169-409X(96)00423-1 11259830

[B26] Liu H. Ye F. Sun Q. Liang H. Li C. Li S. (2021). *Scutellaria baicalensis* extract and baicalein inhibit replication of SARS-CoV-2 and its 3C-like protease in vitro. *J. Enzyme Inhib. Med. Chem.* 36 497–503. 10.1080/14756366.2021.1873977 33491508 PMC7850424

[B27] Liu Z.-W. Luo Z.-H. Meng Q.-Q. Zhong P.-C. Hu Y.-J. Shen X.-L. (2020). Network pharmacology-based investigation on the mechanisms of action of *Morinda officinalis* How. in the treatment of osteoporosis. *Comput. Biol. Med.* 127:104074. 10.1016/j.compbiomed.2020.104074 33126122

[B28] Lyu M. Fan G. Xiao G. Wang T. Xu D. Gao J. (2021). Traditional Chinese medicine in COVID-19. *Acta Pharm. Sin. B* 11 3337–3363. 10.1016/j.apsb.2021.09.008 34567957 PMC8450055

[B29] Muegge I. Heald S. L. Brittelli D. (2001). Simple selection criteria for drug-like chemical matter. *J. Med. Chem.* 44 1841–1846. 10.1021/jm015507e 11384230

[B30] Pan H.-D. Yao X.-J. Wang W.-Y. Lau H.-Y. Liu L. (2020). Network pharmacological approach for elucidating the mechanisms of traditional Chinese medicine in treating COVID-19 patients. *Pharmacol. Res.* 159:105043. 10.1016/j.phrs.2020.105043 PMC7305506 32569819

[B31] Qiao X. Li R. Song W. Miao W.-J. Liu J. Chen H.-B. (2016). A targeted strategy to analyze untargeted mass spectral data: rapid chemical profiling of *Scutellaria baicalensis* using ultrahigh performance liquid chromatography coupled with hybrid quadrupole orbitrap mass spectrometry and key ion filtering. *J. Chromatogr. A* 1441 83–95. 10.1016/j.chroma.2016.02.079 26952367

[B32] Ren Y. Liang S. Zheng Y. Deng X. Lei L. Ai J. (2021). Investigation on the function tropism of Tiaoqin and Kuqin (different specification of *Scutellaria baicalensis*) by comparing their curative effect on different febrile disease model. *J. Ethnopharmacol.* 268:113596. 10.1016/j.jep.2020.113596 33221498

[B33] Ru J. Li P. Wang J. Zhou W. Li B. Huang C. (2014). TCMSP: a database of systems pharmacology for drug discovery from herbal medicines. *J. Cheminf.* 6 1–6. 10.1186/1758-2946-6-13 24735618 PMC4001360

[B34] Safran M. Dalah I. Alexander J. Rosen N. Iny Stein T. Shmoish M. (2010). GeneCards version 3: the human gene integrator. *Database* 2010:baq020. 10.1093/database/baq020 20689021 PMC2938269

[B35] Shannon P. Markiel A. Ozier O. Baliga N. S. Wang J. T. Ramage D. (2003). Cytoscape: a software environment for integrated models of biomolecular interaction networks. *Genome Res.* 13 2498–2504. 10.1101/gr.1239303 14597658 PMC403769

[B36] Song J. Zhang L. Xu Y. Yang D. Yang S. Zhang W. (2021). The comprehensive study on the therapeutic effects of baicalein for the treatment of COVID-19 in vivo and in vitro. *Biochem. Pharmacol.* 183:114302. 10.1016/j.bcp.2020.114302 33121927 PMC7588320

[B37] Song J.-W. Long J.-Y. Xie L. Zhang L.-L. Xie Q.-X. Chen H.-J. (2020). Applications, phytochemistry, pharmacological effects, pharmacokinetics, toxicity of *Scutellaria baicalensis* Georgi. and its probably potential therapeutic effects on COVID-19: a review. *Chin. Med.* 15 1–26. 10.1186/s13020-020-00384-0 32994803 PMC7517065

[B38] Szklarczyk D. Gable A. L. Nastou K. C. Lyon D. Kirsch R. Pyysalo S. (2021). The STRING database in 2021: customizable protein–protein networks, and functional characterization of user-uploaded gene/measurement sets. *Nucleic Acids Res.* 49 D605–D612. 10.1093/nar/gkaa1074 33237311 PMC7779004

[B39] Teli D. M. Shah M. B. Chhabria M. T. (2021). In silico screening of natural compounds as potential inhibitors of SARS-CoV-2 main protease and spike RBD: targets for COVID-19. *Front. Mol. Biosci.* 7:599079. 10.3389/fmolb.2020.599079 33542917 PMC7852456

[B40] Tsugawa H. Cajka T. Kind T. Ma Y. Higgins B. Ikeda K. (2015). MS-DIAL: data-independent MS/MS deconvolution for comprehensive metabolome analysis. *Nat. Methods* 12 523–526. 10.1038/nmeth.3393 25938372 PMC4449330

[B41] Veber D. F. Johnson S. R. Cheng H.-Y. Smith B. R. Ward K. W. Kopple K. D. (2002). Molecular properties that influence the oral bioavailability of drug candidates. *J. Med. Chem.* 45 2615–2623. 10.1021/jm020017n 12036371

[B42] Xu H.-Y. Zhang Y.-Q. Liu Z.-M. Chen T. Lv C.-Y. Tang S.-H. (2019). ETCM: an encyclopaedia of traditional Chinese medicine. *Nucleic Acids Res.* 47 D976–D982. 10.1093/nar/gky987 30365030 PMC6323948

[B43] Xu T. Meng J.-R. Cheng W. Liu J.-Z. Chu J. Zhang Q. (2022). Discovery of honokiol thioethers containing 1, 3, 4-oxadiazole moieties as potential α-glucosidase and SARS-CoV-2 entry inhibitors. *Bioorg. Med. Chem.* 67:116838. 10.1016/j.bmc.2022.116838 35617790 PMC9123836

[B44] Xu T. Tian W. Zhang Q. Liu J. Liu Z. Jin J. (2021). Novel 1, 3, 4-thiadiazole/oxadiazole-linked honokiol derivatives suppress cancer via inducing PI3K/Akt/mTOR-dependent autophagy. *Bioorg. Chem.* 115:105257. 10.1016/j.bioorg.2021.105257 34426156

[B45] Yang R. Liu H. Bai C. Wang Y. Zhang X. Guo R. (2020). Chemical composition and pharmacological mechanism of Qingfei Paidu decoction and Ma Xing Shi Gan decoction against Coronavirus disease 2019 (COVID-19): in silico and experimental study. *Pharmacol. Res.* 157:104820. 10.1016/j.phrs.2020.104820 32360484 PMC7194979

[B46] Yao T. Yan J. Li Y. Wang J. Qiao M. Hu X. (2020). An integrated approach based on phytochemistry, network pharmacology and metabolomics reveals the mechanism of action of *Xanthium strumarium* L. for allergic rhinitis. *RSC Adv.* 10 41154–41163. 10.1039/D0RA06763F 35519219 PMC9057783

[B47] Zhang Y. Lv P. Ma J. Chen N. Guo H. Chen Y. (2022). *Antrodia cinnamomea* exerts an anti-hepatoma effect by targeting PI3K/AKT-mediated cell cycle progression in vitro and in vivo. *Acta Pharm. Sin. B* 12 890–906. 10.1016/j.apsb.2021.07.010 35256953 PMC8897033

[B48] Zhao Z. Li Y. Zhou L. Zhou X. Xie B. Zhang W. (2021). Prevention and treatment of COVID-19 using traditional Chinese medicine: a review. *Phytomedicine* 85:153308. 10.1016/j.phymed.2020.153308 32843234 PMC7439087

[B49] Zhou H. Zhang Y. Liang H. Song H. Zhao J. Liu L. (2021). A novel multidimensional strategy to evaluate *Belamcanda chinensis* (L) DC and *Iris tectorum* maxim based on plant metabolomics, digital reference standard analyzer and biological activities evaluation. *Chin. Med.* 16 1–18. 10.1186/s13020-021-00494-3 34446058 PMC8393741

[B50] Zhou Y. Zhou B. Pache L. Chang M. Khodabakhshi A. H. Tanaseichuk O. (2019). Metascape provides a biologist-oriented resource for the analysis of systems-level datasets. *Nat. Commun.* 10 1–10. 10.1038/s41467-019-09234-6 30944313 PMC6447622

