# Supplementary material for: Integrated network pharmacology analysis, molecular docking, LC-MS analysis and bioassays revealed the potential active ingredients and underlying mechanism of Scutellariae radix for COVID-19
Source: Front Plant Sci. 2022 Sep 15;13:988655. doi: 10.3389/fpls.2022.988655 (PMC9520067; doi:10.3389/fpls.2022.988655)
Supplement: Supplementary file 1 [file Data_Sheet_1.docx]

**Supporting Information for**

**Original articles**

**Integrated Network Pharmacology Analysis, Molecular Docking, LC-MS Analysis and Bioassays Revealed the Potential Active Ingredients and Underlying Mechanism of *Scutellariae Radix* for COVID-19**

**Jiazheng Liu^1†^, Jieru Meng^1†^, Runfeng Li^2†^, Haiming Jiang^2^, Lu Fu^1^, Ting Xu^1^, Guo-Yuan Zhu^1^, Wei Zhang^1^, Jin Gao^4^, Zhi-Hong Jiang^1^*, Zi-Feng Yang^2,3*^, Li-Ping Bai^1*^**

^1^ *State Key Laboratory of Quality Research in Chinese Medicine, Macau Institute for Applied Research in Medicine and Health, Guang-dong-Hong Kong-Macao Joint Laboratory of Respiratory Infectious Disease, Macau University of Science and Technology, Taipa, Macau, People’s Republic of China.*

^2^ *State Key Laboratory of Respiratory Disease, National Clinical Research Center for Respiratory Disease, Guangzhou Institute of Respiratory Health，the First Affiliated Hospital of Guangzhou Medical University, Guangzhou, Guangdong, 510000, P. R. China.*

*^3^ Guangzhou Laboratory; Guangzhou Key Laboratory for Clinical Rapid Diagnosis and Early Warning of Infectious Diseases, Guangzhou, Guangdong, 510000, P. R. China.*

^4^IncreasePharm (Hengqin) Institute Co., Ltd, Zhu Hai, Guangdong 519031, People’s Republic of China.

†These authors contributed equally to this work and share first authorship

*** Correspondence:**

Zhi-Hong Jiang: lzhjiang@must.edu.mo

Zi-Feng Yang: jeffyah@163.com

Li-Ping Bai: lpbai@must.edu.mo


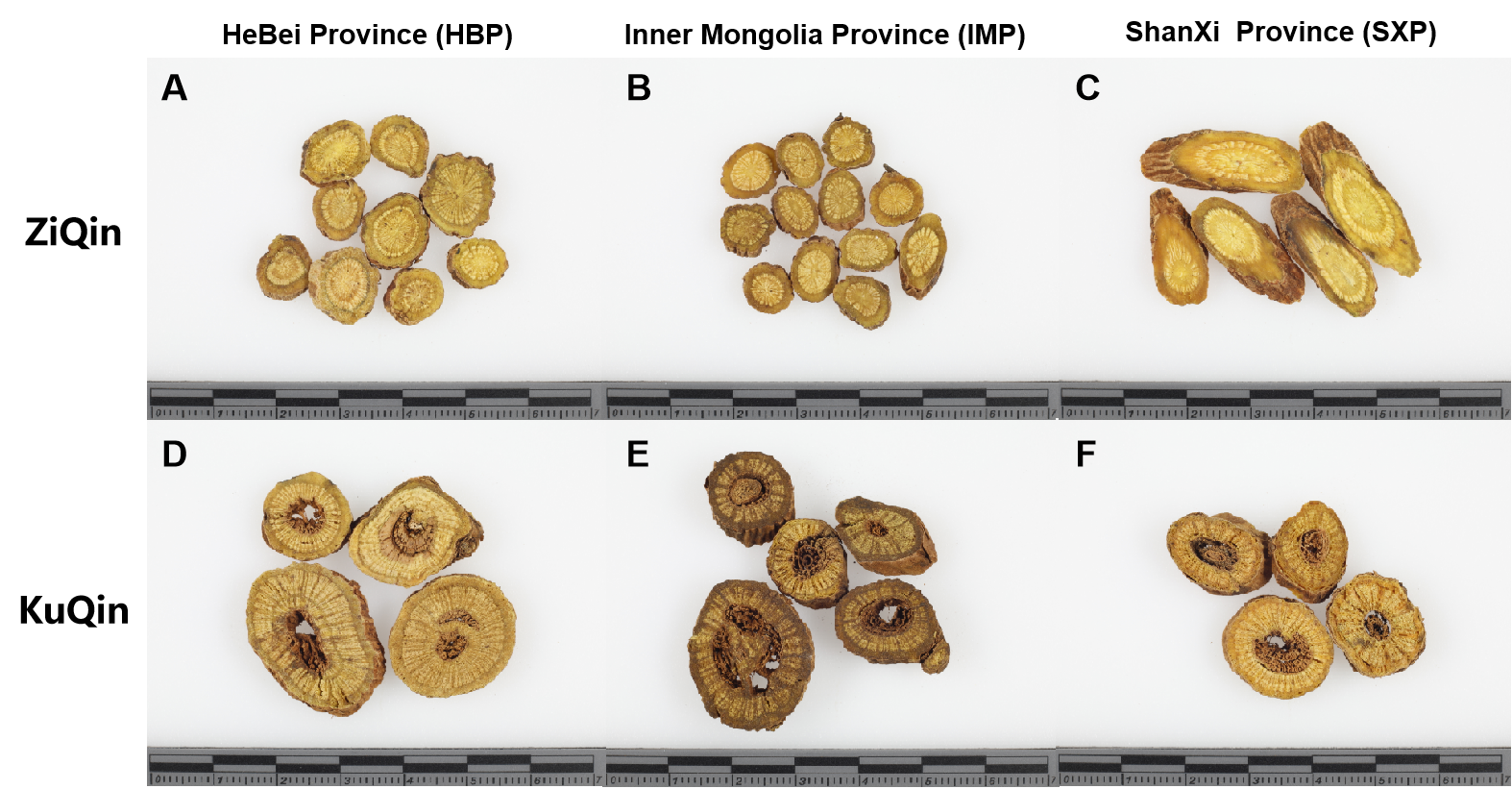


**Figure S1** Representative samples of ZiQin and KuQin

**
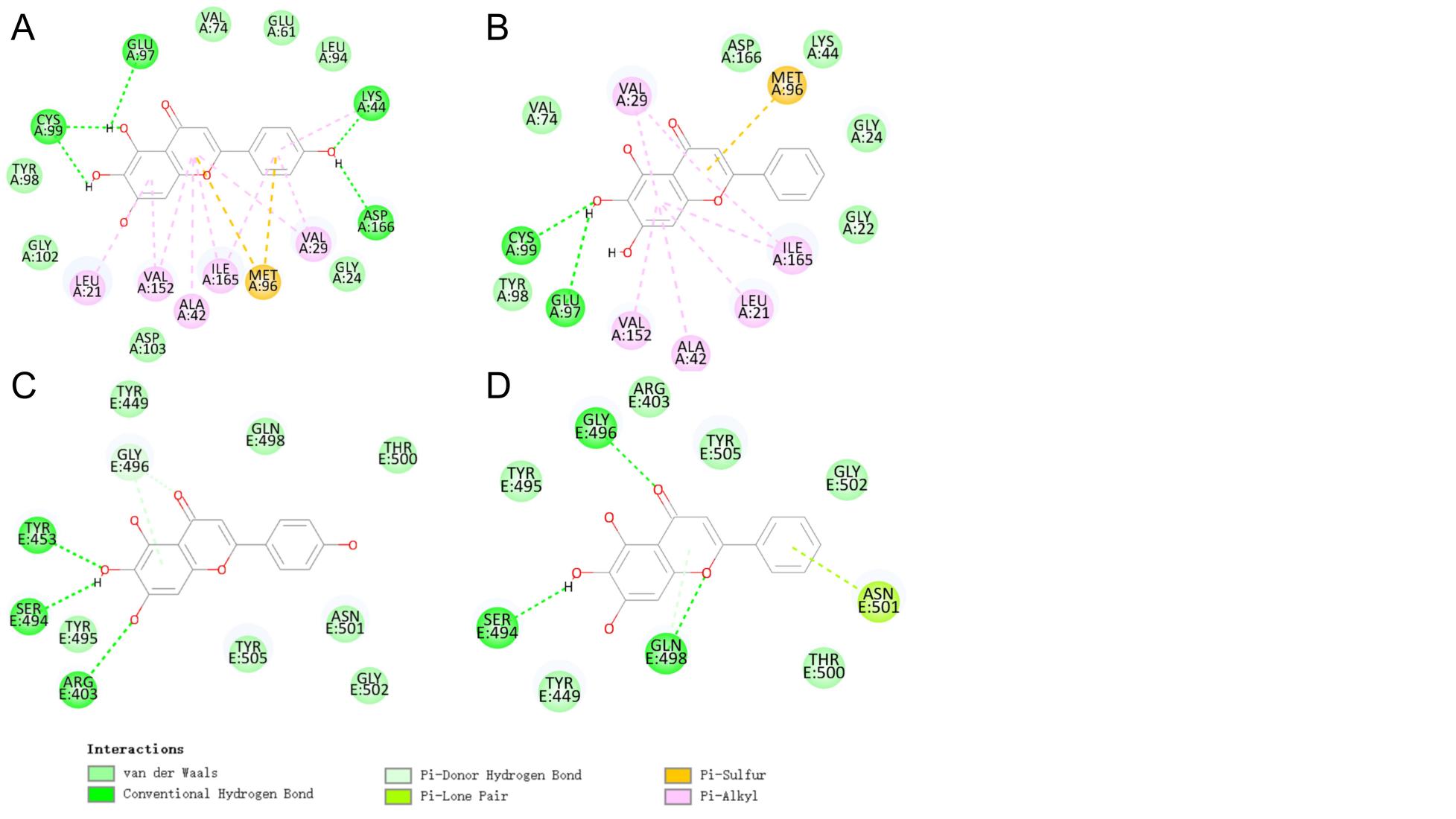
**

**Figure S2** Molecular models of scutellarein and baicalein binding to the predicted pivotal targets. (A) Scutellarein with IKBKB. (B) Baicalein with IKBKB. (C) Scutellarein with SARS-CoV-2 Spike. (D) Baicalein with SARS-CoV-2 Spike.

**
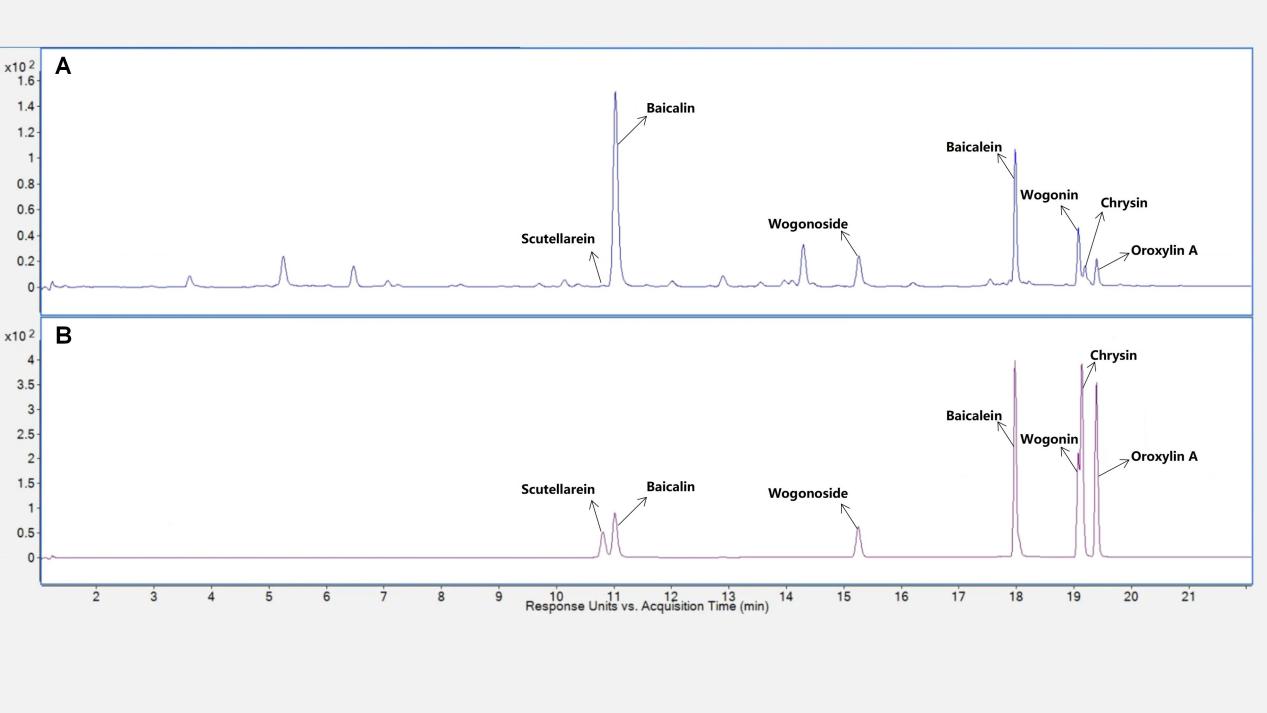
**

**Figure S3** Chemical identification confirmed by mixed standards. (A) Representative UHPLC-UV chromatogram of *Scutellariae Radix* extract. (B) UHPLC-UV chromatogram of mixed standards under wavelength of 270 nm.


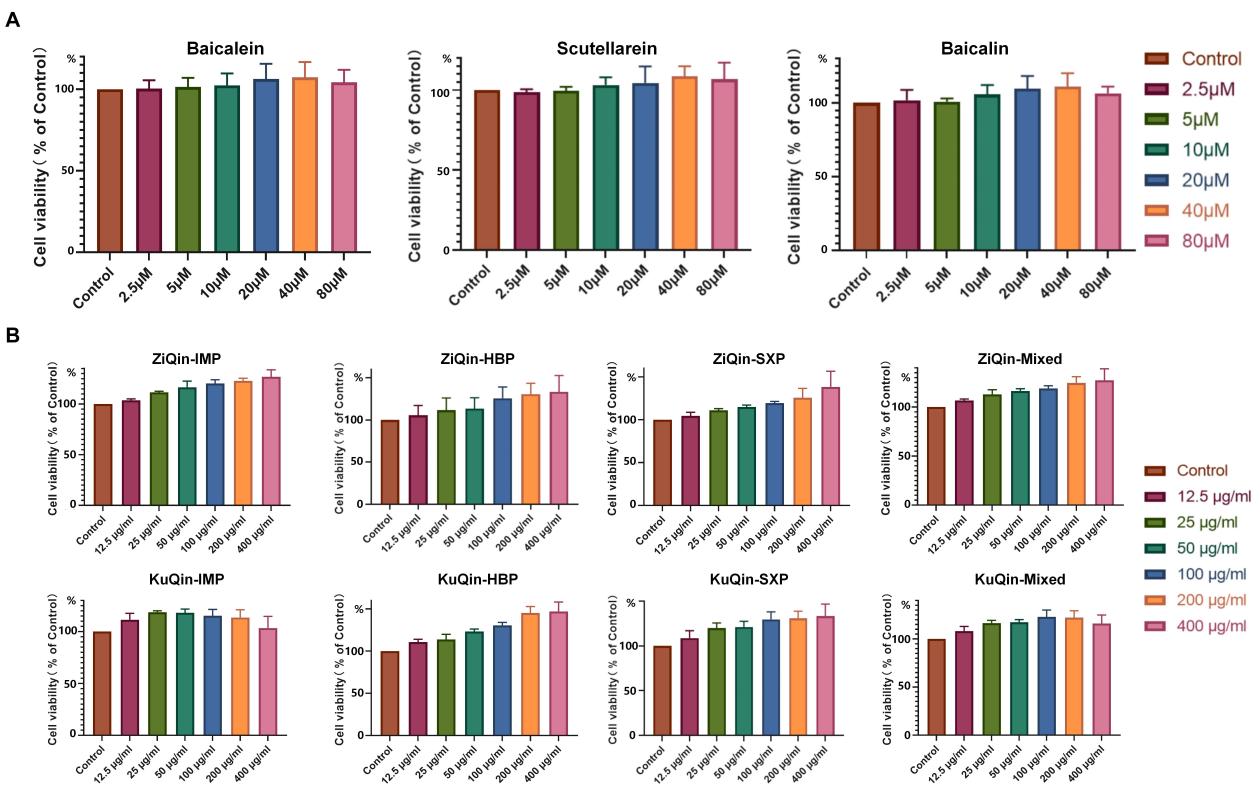


**Figure S4** Cytotoxicity of different extracts of *Scutellariae Radix* and its components on RAW264.7 cells. (A) Cytotoxicity of baicalein, scutellarein, and baicalin on RAW264.7 cells. The cells were treated with different concentrations of compounds (2.5 - 80 μM) for 24h. The viability was analyzed with MTT assay. (B) Cytotoxicity of ZiQin extracts and KuQin extracts on RAW264.7 cells with indicated concentrations by MTT method.

**Table S1** Sample information of ZiQin and KuQin.

| Name | Growth origin | Longitude and latitude | Collected time | Supplier |
| --- | --- | --- | --- | --- |
| ZiQin | Inner Mongolia Province, China | 111.765617; 40.817498 | September 2021 | Beijing Tong Ren Tang Group, China |
| ZiQin | HeBei Province, China | 114.468664; 38.037057 | December 2020 | Zhuhai jiabaohua health pharmacy Ltd, China |
| ZiQin | ShanXi Province, China | 112.549248; 37.857014 | March 2021 | Dashenlin Pharmaceutical Group Ltd, China |
| KuQin | Inner Mongolia Province, China | 111.765617; 40.817498 | October 2020 | JingFangJi Pharmacy, China |
| KuQin | HeBei Province, China | 114.468664; 38.037057 | November 2020 | WenWuShan Pharmacy, China |
| KuQin | ShanXi Province, China | 112.549248; 37.857014 | November 2020 | China Nepstar Chain Drugstore Ltd, China |

**Table S2 - Table S9 were provided in the supplementary Excel file (Supplementary materials 2).**

**Table S10** Quantitative analysis results of baicalin and baicalein.

| Compound | Sample name | Content (mg/g) |
| --- | --- | --- |
| Baicalin | KQ-IMP-1 | 6.29 |
|  | KQ-IMP-2 | 6.20 |
|  | KQ-IMP-3 | 6.09 |
|  | KQ-HBP-1 | 4.95 |
|  | KQ-HBP-2 | 4.93 |
|  | KQ-HBP-3 | 5.07 |
|  | KQ-SXP-1 | 6.26 |
|  | KQ-SXP-2 | 6.22 |
|  | KQ-SXP-3 | 6.19 |
|  | KQ-Mixed-1 | 5.67 |
|  | KQ-Mixed-2 | 5.65 |
|  | KQ-Mixed-3 | 5.91 |
|  | ZQ-IMP-1 | 7.78 |
|  | ZQ-IMP-2 | 8.35 |
|  | ZQ-IMP-3 | 7.91 |
|  | ZQ-HBP-1 | 3.25 |
|  | ZQ-HBP-2 | 3.42 |
|  | ZQ-HBP-3 | 3.36 |
|  | ZQ-SXP-1 | 3.52 |
|  | ZQ-SXP-2 | 3.54 |
|  | ZQ-SXP-3 | 3.65 |
|  | ZQ-Mixed-1 | 4.55 |
|  | ZQ-Mixed-1 | 4.62 |
|  | ZQ-Mixed-1 | 4.74 |
| Baicalein | KQ-IMP-1 | 5.65 |
|  | KQ-IMP-2 | 5.64 |
|  | KQ-IMP-3 | 5.57 |
|  | KQ-HBP-1 | 4.13 |
|  | KQ-HBP-2 | 4.03 |
|  | KQ-HBP-3 | 4.17 |
|  | KQ-SXP-1 | 3.25 |
|  | KQ-SXP-2 | 3.05 |
|  | KQ-SXP-3 | 3.09 |
|  | KQ-Mixed-1 | 4.29 |
|  | KQ-Mixed-2 | 4.36 |
|  | KQ-Mixed-3 | 4.27 |
|  | ZQ-IMP-1 | 1.25 |
|  | ZQ-IMP-2 | 1.21 |
|  | ZQ-IMP-3 | 1.23 |
|  | ZQ-HBP-1 | 1.29 |
|  | ZQ-HBP-2 | 1.22 |
|  | ZQ-HBP-3 | 1.14 |
|  | ZQ-SXP-1 | 0.62 |
|  | ZQ-SXP-2 | 0.69 |
|  | ZQ-SXP-3 | 0.63 |
|  | ZQ-Mixed-1 | 1.13 |
|  | ZQ-Mixed-2 | 1.05 |
|  | ZQ-Mixed-3 | 1.15 |
| Baicalin | KQ(Mean value±SD) | 5.79±0.53 |
|  | ZQ(Mean value±SD) | 4.89±1.96 |
| Baicalin | KQ(Mean value±SD) | 4.29±0.92 |
|  | ZQ(Mean value±SD) | 1.05±0.24 |
